# Disruption in glutathione metabolism and altered energy production in the liver and kidney after ischemic acute kidney injury in mice

**DOI:** 10.1038/s41598-024-64586-4

**Published:** 2024-06-15

**Authors:** Peter R. Baker, Amy S. Li, Benjamin R. Griffin, Hyo-Wook Gil, David J. Orlicky, Benjamin M. Fox, Bryan Park, Genevieve C. Sparagna, Jared Goff, Christopher Altmann, Hanan Elajaili, Kayo Okamura, Zhibin He, Daniel Stephenson, Angelo D’Alessandro, Julie A. Reisz, Eva S. Nozik, Carmen C. Sucharov, Sarah Faubel

**Affiliations:** 1 Division of Clinical Genetics and Metabolism, Department of Pediatrics, University of Colorado Anschutz Medical Campus, 13123 East 16th Avenue, Box 300, Aurora, CO 80045 USA; 2 Division of Renal Diseases and Hypertension, Department of Internal Medicine, University of Colorado Anschutz Medical Campus, Mail Stop C281, 12700 East 19th Avenue, Aurora, CO 80045 USA; 3 Division of Nephrology, Department of Medicine, University of Iowa Carver College of Medicine, Iowa City, IA 52242 USA; 4 Department of Internal Medicine, Soonchunhyang University Cheonan Hospital, Cheonan, Republic of Korea; 5 Department of Pathology, University of Colorado Anschutz Medical Campus, 12700 East 19th Avenue, Aurora, CO 80045 USA; 6 Division of Pulmonary Sciences and Critical Care, Department of Internal Medicine, University of Colorado Anschutz Medical Campus, 12700 East 19th Avenue, Aurora, CO 80045 USA; 7 Division of Cardiology, Department of Internal Medicine, University of Colorado Anschutz Medical Campus, 12700 East 19th Avenue, Aurora, CO 80045 USA; 8 Division of Pediatric Critical Care, Department of Pediatrics, University of Colorado Anschutz Medical Campus, 12700 E 19th Ave, Aurora, CO B13180045 USA; 9 Department of Biochemistry and Molecular Genetics, University of Colorado Anschutz Medical Campus, 12801 East 17th Avenue, Aurora, CO 80045 USA

**Keywords:** Acute kidney injury, Metabolomics, Liver

## Abstract

Acute kidney injury (AKI) is a systemic disease that affects energy metabolism in various remote organs in murine models of ischemic AKI. However, AKI-mediated effects in the liver have not been comprehensively assessed. After inducing ischemic AKI in 8–10-week-old, male C57BL/6 mice, mass spectrometry metabolomics revealed that the liver had the most distinct phenotype 24 h after AKI versus 4 h and 7 days. Follow up studies with in vivo [^13^C_6_]-glucose tracing on liver and kidney 24 h after AKI revealed 4 major findings: (1) increased flux through glycolysis and the tricarboxylic (TCA) cycle in both kidney and liver; (2) depleted hepatic glutathione levels and its intermediates despite unchanged level of reactive oxygen species, suggesting glutathione consumption exceeds production due to systemic oxidative stress after AKI; (3) hepatic ATP depletion despite unchanged rate of mitochondrial respiration, suggesting increased ATP consumption relative to production; (4) increased hepatic and renal urea cycle intermediates suggesting hypercatabolism and upregulation of the urea cycle independent of impaired renal clearance of nitrogenous waste. Taken together, this is the first study to describe the hepatic metabolome after ischemic AKI in a murine model and demonstrates that there is significant liver-kidney crosstalk after AKI.

## Introduction

Acute kidney injury (AKI) is a common complication that occurs in approximately 20% of hospitalized patients^1^ and is associated with increased mortality, regardless of whether renal replacement therapy (RRT) is required. The systemic effects of AKI that may contribute to mortality have been extensively investigated in preclinical models. Clinical and preclinical studies have revealed that AKI is a complex systemic disease with effects on virtually every organ^2^, including the lung^3,4^, heart^5,6^, liver^7,8^, brain^9^, and intestine^10^. Reducing the significant mortality of AKI will require addressing these systemic complications^5,11, 12^.

An important systemic consequence of AKI is its effects on energy metabolism and redox balance. Clinically, AKI has long been considered a hypercatabolic state, which adversely affects outcomes, including mortality^13,14^. Metabolomic studies in the preclinical ischemic AKI model have shed substantial light on this issue and demonstrated widespread changes in energy metabolism in the kidney as well as remote organs including the heart and lung^6,15, 16^. In the heart, the metabolome is characterized by amino acid depletion, evidence of altered redox balance including glutathione depletion, and a shift from oxidative phosphorylation to alternative energy production pathways; these metabolic changes are associated with a 50% reduction in cardiac adenosine triphosphate (ATP) levels and impaired cardiac function^6^. Similar changes have been observed in the lung which is also characterized by evidence of increased oxidative stress including glutathione depletion, a shift to alternative methods of energy production, and reduced levels of ATP^15^. These in-organ changes in energy metabolism are also reflected in the plasma metabolome after ischemic AKI, which is characterized by the depletion of numerous amino acids and energy substrates^6^. Thus, energy deficiency, depleted levels of energy substrates, and glutathione depletion are widespread systemic features of AKI.

Although the liver is a major metabolic organ, the effect of AKI on the hepatic metabolome has not previously been examined to our knowledge. Therefore, in the present study, we sought to characterize the liver metabolome after ischemic AKI. We hypothesized that the hepatic metabolome would be similarly characterized by increased alternative energy pathway utilization, altered redox balance and glutathione depletion, and reduced levels of ATP.

## Materials and methods

### Animals

Adult (8–10‐week‐old), male C57BL/6 mice (Jackson Laboratories, Bar Harbor, ME) weighing between 20 and 25 g were used. Mice were maintained on a standard diet, and water was freely available. All experiments were conducted with adherence to the National Institutes of Health Guide for the Care and Use of Laboratory Animals. The animal protocol was approved by the Animal Care and Use Committee of the University of Colorado, Denver. All experiments were performed following the ARRIVE guidelines (http://arriveguidelines.org) to report animal experiments.

### Surgical protocol

To induce ischemic AKI^17^, mice were anesthetized with intraperitoneal avertin (2,2,2-tribromoethanol; Sigma Aldrich, Milwaukee, WI), a laparotomy was performed, and both renal pedicles were clamped for 22 min. Mice received 500 µl saline with buprenorphine subcutaneous injection preceding surgery and 500 µl saline was administered by subcutaneous injection daily after surgery. The sham procedure is similar and consists of laparotomy without renal pedicle clamping. At each post-operative time point of interest, mice were then euthanized with intraperitoneal injection of pentobarbital.

### Collection and preparation of plasma, liver, and kidney specimens for metabolomics

Blood was obtained via cardiac puncture and centrifuged at 3000 g at 4 °C for 10 min; plasma was collected and centrifuged a second time at 3000 g for 1 min. For metabolomics studies, the liver lobes were collected, weighed, snap frozen in liquid nitrogen, and then stored at −80 °C; in order to limit time to freezing (and potential changes in metabolic phenotype due to death) no additional processing of tissue occurred and organs were not perfused prior to collection. The samples for hepatic metabolomics analysis were part of a larger metabolomics study of the plasma, kidney, heart, and lung—data from which have been reported^6,15^.

### Sample processing for metabolomics

Frozen liver and kidney samples were weighed and transferred to 1.5 mL Safelock Eppendorf tubes. Glass homogenization beads were added to each tube followed by cold lysis buffer (MeOH:MeCN:H_2_O (5:3:2, v:v:v) to a final concentration of 15 mg/mL in each sample. Tissue samples were then placed in a homogenizer (Bulletblender Storm 24) for 5 min at 4 °C at intensity level 6. Samples were agitated via vortex at 4 °C for 30 min then centrifuged at 18,213 g for 10 min at 4 °C. Protein and lipid pellets were discarded, while 10 µL injections of supernatants were analyzed via a Vanquish UHPLC system (Thermo Fisher Scientific) coupled to a Q Exactive mass spectrometer (Thermo Fisher Scientific) utilizing an untargeted acquisition as previously reported^18^. Metabolites were resolved across a 2.1 × 150 mm, 1.7 µm Kinetex C18 column (Phenomenex) using a 5-min, reverse-phase gradient from a previously described method^19^. Run order of samples was randomized and in lieu of internal standards, technical replicates were injected every 8 samples to assess quality control and instrument performance. Raw files were converted to mzXML using RawConverter. The resultant files were processed with El-Maven (Elucidata) alongside the Kyoto Encyclopedia of Genes and Genomes (KEGG)^20–22^ database for metabolite assignment and manual peak integration as previously described using intact mass, isotopic patterns, and an in-house standard library^23^. Raw peak areas were utilized for subsequent analysis and no data points were excluded.

### In vivo [D-^13^C_6_]-glucose administration for stable isotope tracing

[D-^13^C_6_]-glucose (Sigma: #389,374) was dissolved in sterile PBS to prepare 5% (w/v) solution. Renal IRI or sham surgery was performed prior to the glucose tracing experiment. 24 h after the clamp removal, mice (*n* = 8 sham and *n* = 8 AKI) were injected at the dose of 5 ml/kg via tail vein and were sacrificed 30 min after the injection. Blood was obtained via cardiac puncture and centrifuged at 4000 rpm at 4 °C for 10 min to collect plasma. After the blood collection, mice were flushed with ice-cold PBS to remove residual blood. Sections of liver lobes, kidney, left ventricle tissue were collected, snap frozen in liquid nitrogen, and then stored at −80 °C for future analysis. For metabolomics analysis, small pieces of tissue (~ 10 mg) were cut, weighed and collected in separate tubes.

### Serum creatinine and blood urea nitrogen (BUN) assay

Serum creatinine was determined using the Point creatinine assay (#C7548) according to the manufacturer’s directions; serum BUN was determined using the Bioassay systems assay (DIUR-100) according to the manufacturer’s directions.

### Aspartate aminotransferase (AST) and alanine aminotransferase (ALT) assay

Plasma AST and ALT were measured using BioAssay Systems EnzyChrom Alanine Transaminase Assay Kit (cat# EALT-100) and EnzyChrom Aspartate Transaminase Assay Kit (cat# EASTR-100) according to the manufacturer’s instructions. Briefly, plasma was incubated with the kit reagents and absorbance was measured at 340 nm at 5 and 10 min. The rate of NADH consumption was calculated by change in OD reading at 5 min and 10 min.

### ATP assay

ATP was measured as previously described^6,15^ and briefly described here. Pre-weighed liver tissue was processed for determination of liver ATP content using commercially available reagents as per manufacturer’s instructions (Abcam; ab833355). Briefly, flash-frozen liver was homogenized in ATP assay buffer using Dounce homogenizer. The lysate was centrifuged at 13000 g at 4 °C, and supernatant subjected to deproteinization procedure via TCA precipitation (Abcam; ab83355). The deproteinized samples were incubated with necessary reaction components for 30 min at room temperature protected from light. Fluorescence signals from samples were then measured on a microplate reader at Ex/Em = 535/587 nm. Serial dilutions of ATP were used to generate a standard calibration curve. ATP concentrations were calculated from the standard curve data and normalized to corresponding tissue weight.

### Liver histology

Liver tissue was fixed overnight in 10% formaldehyde, transferred to 70% ethanol for 48 h and then processed into paraffin blocks. 4-micron sections were stained with hematoxylin and eosin (H&E). Liver injury, in slides blinded to treatment and grouping, was scored by an experienced histologist (DO) as previously described^24,25^ and included assessment of features of hepatocyte injury, the presence of inflammatory cells and foci, steatosis, and markers of tissue response. The entire piece of liver tissue was examined for pathology (approximately 1 square centimeter).

### Liver myeloperoxidase activity

To determine liver myeloperoxidase (MPO) activity, liver tissue was homogenized in 1 ml of cold hexdecyltrimethylammonium bromide buffer, sonicated on ice for 10 s and centrifuged at 14,000xg for 30 min at 4 °C. Twenty microliters of supernatant was transferred into a 96-well plate, and 200 µl of 37 °C o-dianisidine hydrochloride solution was added immediately before the optical density was read at 450 nm and again 30 s later, as previously described^26^.

### High resolution respirometry

The rate of oxygen consumption in the kidney and liver were measured using the Oroboros O2k system (Oroboros Instruments, Innsbruck, Austria) in animals 24 h after sham or AKI surgeries using a stepwise protocol to evaluate various components of the electron transport system. After removal from the animal, a small piece of tissue was placed in cold BIOPS until homogenized^24,25^. Using a 1:100 ratio of mg tissue to µL KME buffer (20 mM MOPS, 120 mM KCl, and 1 mM EGTA, pH 7.4), tissue was homogenized in 2 s bursts separated by a 2 s rest for a total of 10 s (or 3 cycles) at 16,600 rpm using IKA T25 digital Ultra-Turrax homogenizer. Standard protocols were followed for calibration of the chambers. Substrates were added in a stepwise fashion in the following order (final concentrations) 0.2 mM palmitoyl carnitine, 1 mM malate, 4 mM ADP, 0.4 mM octanoyl carnitine, 5 mM pyruvate, 10 mM glutamate, 10 mM succinate, 1 µM CCCP, 2 µM rotenone, and 5 µM Antimycin A. Oxygen flux rates were normalized to mg of protein added determined with a BCA protein analysis test (Pierce). Analysis was performed using Oroboros Datlab 7 (https://www.oroboros.at/index.php/product/datlab-7/) and further normalization was done to correct for day-to-day variations by normalizing to the average maximal respiration of the sham group for each day.

### Superoxide measurements in liver and kidney by electron paramagnetic resonance (EPR) spectroscopy

Mitochondrial superoxide in liver and kidney tissue was measured by EPR spectroscopy using a mitochondrial targeted probe, 1-hydroxy-4-[2-triphenylphosphonio)-acetamido]-2,2,6,6-tetramethylpiperidine,1-Hydroxy-2,2,6,6-tetramethyl-4-[2-(triphenylphosphonio)acetamido] piperidinium dichloride (mito-TEMPO-H) (Enzo Life Sciences, Farmingdale, NY)^24,25^. This compound is a cyclic hydroxylamine that is oxidized upon the reaction with superoxide to generate a more stable radical called nitroxide. The concentration of nitroxide radical (mito-TEMPO•) following oxidation of (mito-TEMPO-H) was measured in liver and kidney 24 h following AKI. Tissue was homogenized as detailed above for respirometry studies. Following homogenization, 120 µL of tissue homogenate was incubated with 0.2 mM mito-TEMPO-H in Krebs–Henseleit KHB containing 100 µM in 200 µL of total volume for 1 h at 37 °C. 150 µL of the reaction mixture were loaded in PTFE tubing flash froze in liquid nitrogen. EPR measurements were performed at 77 K using the Bruker EMXnano X-band spectrometer. EPR acquisition parameters were as follows: microwave frequency = 9.65 GHz; center field = 3438 G; modulation amplitude = 6.0 G; sweep width = 150 G; microwave power = 0.316 mW; total number of scans = 3; sweep time = 60 s; and time constant = 1.28 ms. mito-TEMPO nitroxide radical concentration was obtained by double integration followed by Spin Count module (Bruker) and expressed as the nitroxide radical per g of protein.

### Statistical analysis (metabolites)

Autoscaling was utilized to normalize data for statistical analysis using MetaboAnalyst 3.0 (available at http://www.metaboanalyst.ca)^27^. Univariate ANOVA (significant threshold *p* < 0.05) was performed on autoscaled data in Metaboanalyst 3.0. Hierarchical clustering analysis was performed on metabolites that were significant by ANOVA using Morpheus (https://software.broadinstitute.org/morpheus); the correlation metric used was the Pearson (*n*-1) correlation with an average linkage method.

Analytes with a significant *p*-value by ANOVA analysis were further analyzed for false discovery rate (FDR) using the Benjamini–Hochberg method, with significant FDR determined as ≤ 0.05^28^. Post-hoc analysis was then performed using each individual time point (4 h, 24 h, 7d) and metabolites found significant by the above ANOVA analysis. Student t-test was used to determine the 2-tailed *p*-value for individual analytes comparing AKI versus sham. Significant analytes were placed into MetaboAnalyst 3.0. Both Metabolite Set Enrichment Analysis and Metabolic Pathway Analyses functions were used to analyze the ANOVA data across time points. In both analyses “compound name” was used to identify and match compounds in the KEGG, Human Metabolome (HMDB), and/or PubChem databases through the MetaboAnalyst freeware. Pathway enrichment was then performed using both the KEGG and Small Molecule Pathway Databases (SMPDB) curated pathway databases. Pathway enrichment was determined significant if the FDR was ≤ 0.05.

Students t-test was utilized to analyze serum creatinine, BUN, AST, ALT, hepatic glutathione, hepatic ATP, and hepatic MPO activity. Oroboros O2k data was assessed for significance using the student t-test for each individual substrate addition.

## Results

### Study design

The metabolomics study design included 10 mice per group for each of the 7 experimental groups, which have been previously published^6,15^: (1) normal (no surgical procedure), (2) 4 h sham, (3) 4 h AKI, (4) 24 h sham, (5) 24 h AKI, (6) 7 day sham, and (7) 7 day AKI. However, 1 mouse in the 7-day sham group died, and 4 mice in the 7-day AKI group died. Thus, the numbers of animals included in the final analysis are as follows: (1) normal: *n* = 10, (2) 4 h sham: *n* = 10, (3) 4 h AKI: *n* = 10, (4) 24 h sham: *n* = 10, (5) 24 h AKI: *n* = 10, (6) 7 day sham: *n* = 9, and (7) 7 day AKI: *n* = 6. The accompanying plasma, kidney, heart, lung metabolomics results have been previously reported^6,15^.

### Glutathione and ATP depletion in the liver are most severe at 24 h after AKI

**Figure 1:**
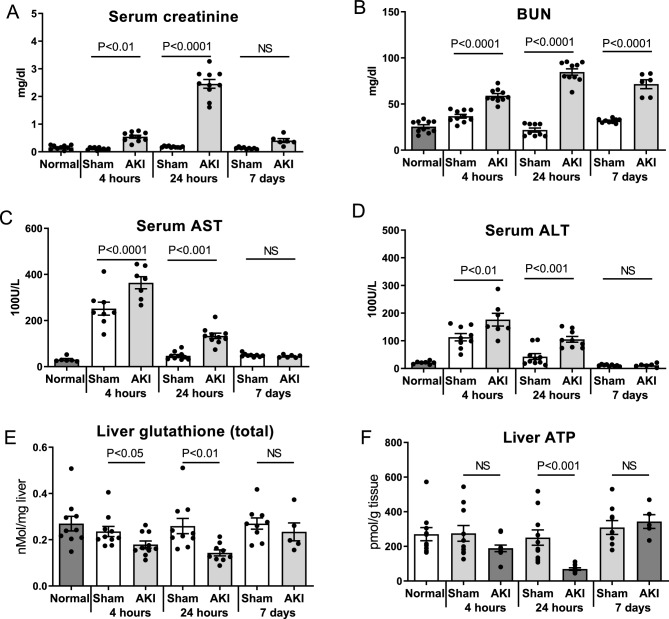
Time course of AKI and hepatic effects after ischemic AKI. Wild type mice were examined 4 h, 24 h, and 7 days after sham surgery (Sham) and surgery to induce ischemic AKI (AKI). Normal mice were also studied. Renal function was assessed by (**A**) Serum creatinine and (**B**) Blood urea nitrogen (BUN) (The serum creatinine and BUN data from this cohort of mice have been previously published)^6,15^. Hepatic effects were assessed by (**C**) Serum AST, (**D**) Serum ALT, (**E**) Liver glutathione (total) levels, and (**F**) Liver ATP. Data are mean ± SE; statistics by Student’s t-test comparing Sham versus AKI at each time point; *n* = 6–10 per group.

To assess kidney function after AKI, serum creatinine and BUN were determined in normal mice and at 4-h, 24-h and 7-days after sham and AKI procedures as previously described^6,16^. As shown in Fig. 1A, serum creatinine was significantly increased 4 and 24 h after AKI versus sham. There was no significant difference in serum creatinine at 7 days. BUN was significantly increased in AKI at all three time points (Fig. 1B). (The serum creatinine and BUN data in this cohort of mice have been previously published^6^.)

Serum AST and ALT, clinical markers of hepatic stress, were significantly increased 4 and 24 h after AKI versus sham (Figs. 1C,D). Liver levels of glutathione, the most abundant antioxidant and an essential cofactor for the enzyme systems that combat reactive oxygen species (ROS), were significantly decreased at 4- and 24-h post-AKI (Fig. 1E), suggestive of increased oxidative stress in the organ. To assess energy production in the liver after AKI, liver ATP levels were determined. Liver ATP levels were trending lower 4 h after AKI and were significantly lower 24 h after AKI (Fig. 1F).

### Hepatic mitochondrial function, superoxide production, and liver histology are normal after AKI

**Figure 2:**
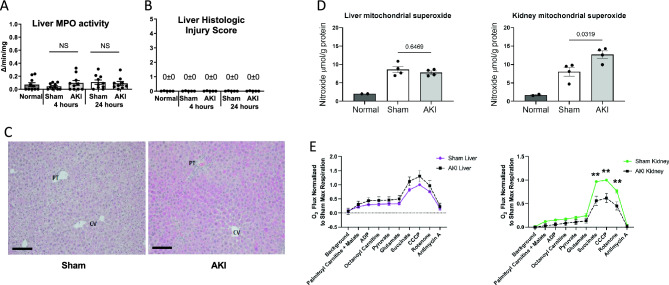
Liver MPO activity, histology, superoxide, and mitochondrial respiration after AKI. To assess whether AKI included liver inflammation, injury, elevated reactive oxygen species (ROS), or mitochondrial dysfunction, (**A**) liver MPO activity, (**B**, **C**) liver histology, (**D**) liver superoxide, and (**E**) mitochondrial respiration were assessed in the liver and were unchanged versus sham in all cases. In contrast, kidney mitochondrial superoxide increased, and kidney mitochondrial respiration was suppressed. Mitochondrial superoxide was assessed with electron paramagnetic resonance (EPR) spectroscopy. Mitochondrial function (O_2_ flux) was measured by the Oroboros O2K respirometer and the substrates added to the chamber are listed. Significant differences between mitochondrial oxygen consumption were found in the kidney using the substrates succinate, CCCP and rotenone, indicating a defect in complex II during AKI. Data are mean ± SE; statistics by Student’s t-test comparing Sham versus AKI at each time point; *n* = 6–10 per group, **p < 0.01.

Since inflammation is associated with oxidative stress and reduced hepatic levels of glutathione, we assessed liver myeloperoxidase (MPO) activity; MPO activity is a sensitive marker of neutrophil infiltration. Liver MPO activity at 4 and 24 h was similar after sham and AKI, and not statistically significant compared to normal mice (Fig. 2A) (liver MPO activity at 7 days was similarly not statistically significant after sham or AKI—data not shown).

To further assess the effect of AKI on the liver, we examined liver histology, which was scored using a composite injury score that included inflammation, cell injury (including necrosis), steatosis, and fibrosis. As shown in Fig. 2B, both sham and AKI scored 0 ± 0 for histologic injury at 4 and 24 h post AKI. Representative histology for sham and AKI at 24 h is shown in Fig. 2C. Slight reactive changes were noted for two of the eight AKI samples as shown in Supplementary Fig. S1). These reactive changes may be associated with liver “stress”, but are not indicative of cell injury, cell death, or inflammation.

Since liver inflammation and injury were absent, we examined hepatic levels of superoxide, the most abundant reactive oxygen species (ROS), to assess whether elevated levels could explain the reduced levels of hepatic glutathione. As shown in Fig. 2D, mitochondrial superoxide was similar in the liver after sham and AKI at 24 h (similar results were observed in the liver 4 h after AKI, data not shown). In contrast, superoxide levels were significantly increased in the kidney 24 h after AKI.

To investigate whether mitochondrial dysfunction could explain the reduced levels of glutathione and ATP in the liver, we assessed mitochondrial respiration. As shown in Fig. 2E, mitochondrial respiration was normal in the liver 24 h after AKI. In contrast, mitochondrial respiration was significantly suppressed in the kidney.

In sum, these data indicate that our model of ischemic AKI is associated with hepatic stress as judged by elevated liver enzymes, depletion of glutathione, and reduced ATP levels. Remarkably, these effects occur in the absence of histologic liver injury, hepatic cell death, neutrophil or other inflammatory cell accumulation, excess ROS, or abnormal mitochondrial function.

### Metabolomic analysis and hierarchical clustering analysis of the liver

To characterize the effect of ischemic AKI on energy metabolism, untargeted UHPLC-MS-based metabolomics analysis of the liver was performed and resulted in the measurement of 141 annotated metabolites in central energy metabolism. Univariate ANOVA (without post hoc testing) revealed that 100 (71%) of the original 141 metabolites were significantly different among experimental groups. Analyte F-value, *P*-value, and Benjamini–Hochberg FDR can be found in Supplementary Table S1. Analytes with the most significant ANOVA corrected *p*-values were 2',3'-cyclic CMP, phosphocreatine, glucuronic acid, creatine, citrulline, ethanolamine phosphate, allantoin, and pantothenate. A principal component analysis (PCA) of all liver samples from untargeted metabolomics, including normal, sham, and AKI at 4 h, 24 h, and 7 days after surgery as well as quality control samples and blank controls can be found in Supplementary Fig. S2.

**Figure 3:**
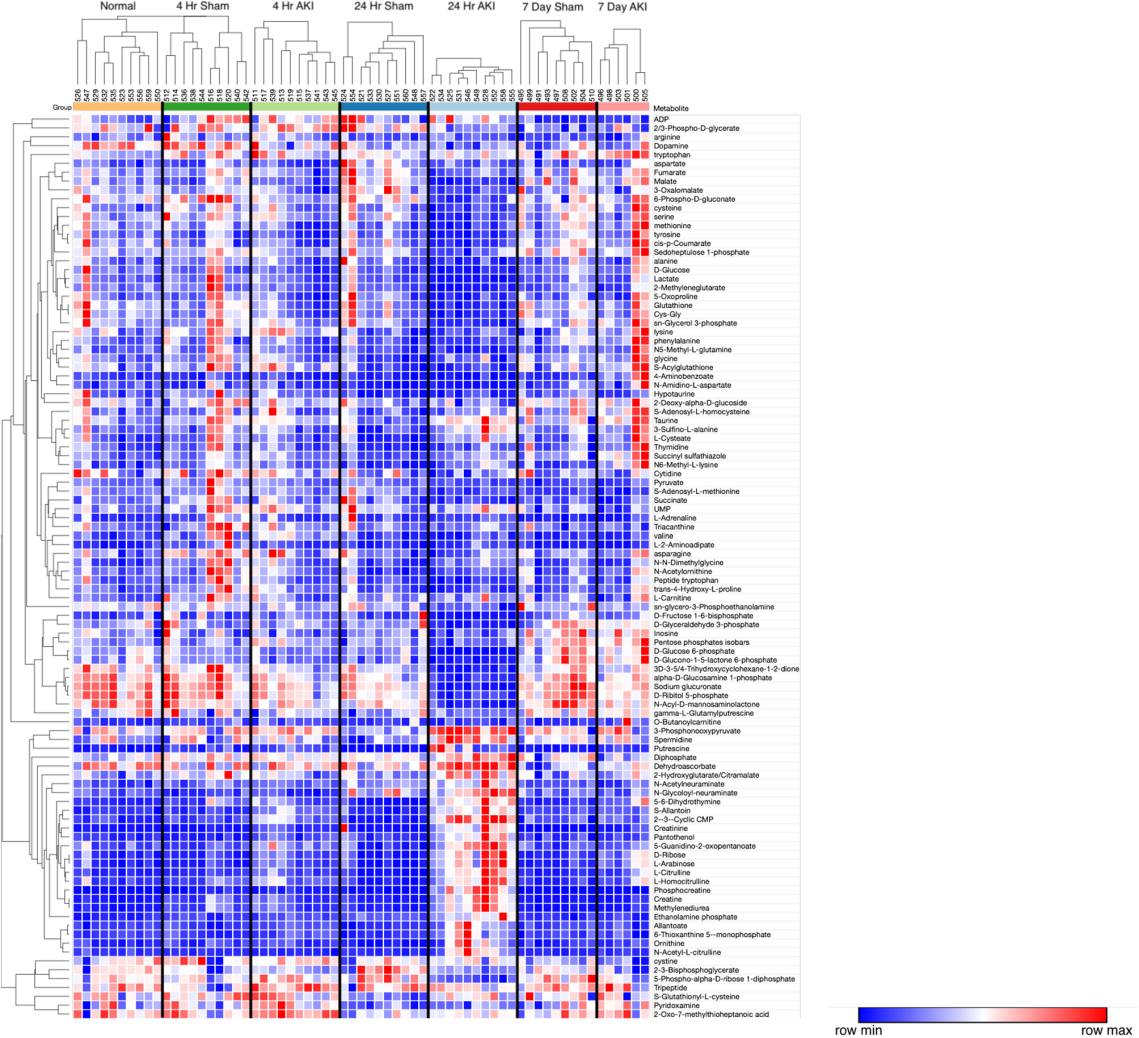
Hierarchical clustering analysis (HCA) of liver metabolites. HCA with predefined sample clusters, was performed for the 102 metabolites that were significantly different among groups as judged by ANOVA (without post hoc testing) to determine trends for individual metabolites among experimental groups. Each column cluster represents an experimental cohort as labeled (Normal, 4-h sham, 4-h AKI, 24-h sham, 24-h AKI, 7-day sham and 7-day AKI), whereas each individual column represents an experimental mouse subject (Normal: *n* = 10, 2 4 h sham: *n* = 10, 4 h AKI: *n* = 10, 24 h sham: *n* = 10, 24 h AKI: *n* = 10, 7 day sham: *n* = 9, and 7 day AKI: *n* = 6). Each row represents a metabolite. To the right of each row is the corresponding metabolite name. Red represents increased metabolite level whereas blue represents decreased metabolite level. The correlation metric used was the Pearson (n-1) correlation with an average linkage method. The major effect observed is for the 24-h AKI group which is characterized by metabolites that are both relatively decreased versus other groups (in blue, upper 2/3 of heat map) as well as increased levels versus other groups (in red, lower 1/3 of heat map).

To identify and visualize trends among experimental groups, these 100 metabolites are presented via hierarchical clustering analysis (HCA) (Fig. 3). Broadly speaking, the HCA heat map demonstrates that the 24-h AKI group has the most distinct metabolic phenotype with approximately 2/3 of the metabolites relatively decreased compared to other groups and approximately 1/3 of the metabolites relatively increased compared to other groups.

Only 5 analytes were significantly different at all three time points (i.e., 4 h, 24 h, and 7 days). All higher in AKI, these included citrulline, allantoin, ribose, arabinose, and (as expected in AKI) creatinine. Comparisons were then made between sham and AKI for each time point separately: 15% (*n* = 21/141) were significantly different at 4 h; 52% (*n* = 73/141) were significantly different at 24 h, and 23% (*n* = 33/141) were significantly different at 7 days. Thus, most analytes with significantly different concentrations were found at the 24-h time point, consistent with the HCA heatmap.

### Pathway enrichment analysis demonstrates that energy metabolism, the urea cycle, and glutathione metabolism are impacted in the liver after AKI

**Figure 4:**
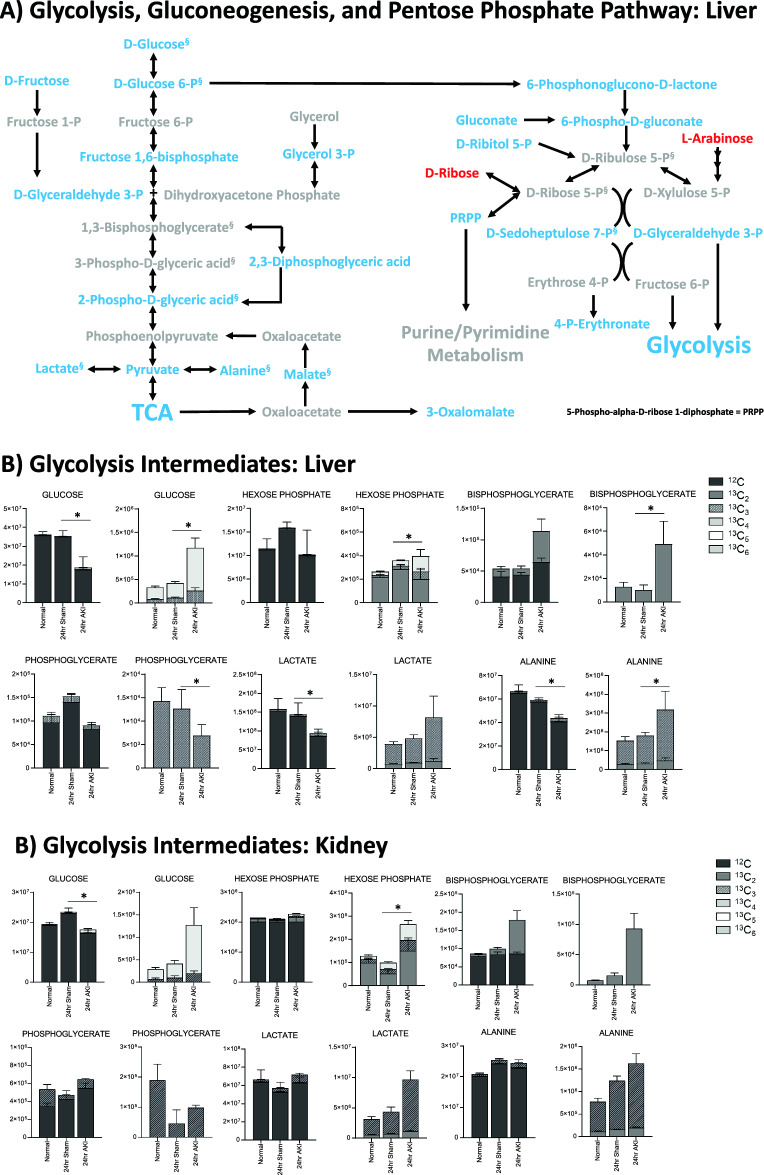

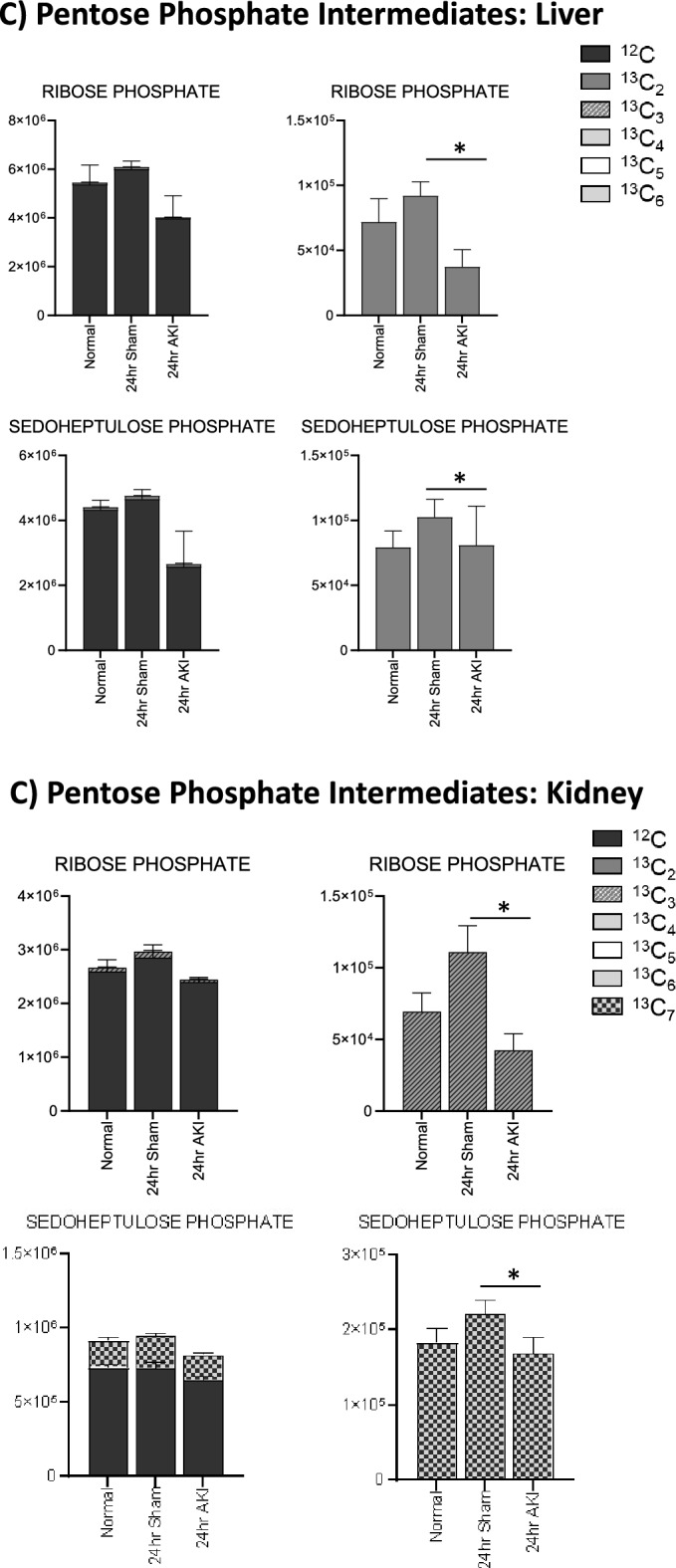
Pathway enrichment and flux analysis reveals that glycolysis, gluconeogenesis, and pentose phosphate pathway in the liver and kidney are impacted 24 h after AKI. (**A**) Pathway diagram including Glycolysis, Gluconeogenesis, and Pentose Phosphate Pathway, illustrating steady state and labeled carbon study results in liver. Metabolites higher (red) or lower (blue) in AKI versus Sham mouse livers at 24 h post-procedure. Analytes in gray were detectable but not significantly different in AKI versus Sham. Analytes in black were not detectable through metabolomic analysis. Differences found in labeled carbon studies are designated by §. (**B**) Labeled carbon study results for Glycolysis and Gluconeogenesis intermediates in liver and kidney. (**C**) Labeled carbon study results for Pentose Phosphate Pathway intermediates in liver and kidney. For (**B**) & (**C**), *y*-axes represent peak areas (arbitrary units). For both liver and kidney, *n* = 8 Sham, *n* = 8 AKI. *P*-value < 0.05 (*) in Sham vs AKI. Data can be found in Supplementary Tables S6 and S7, respectively.

**Figure 5:**
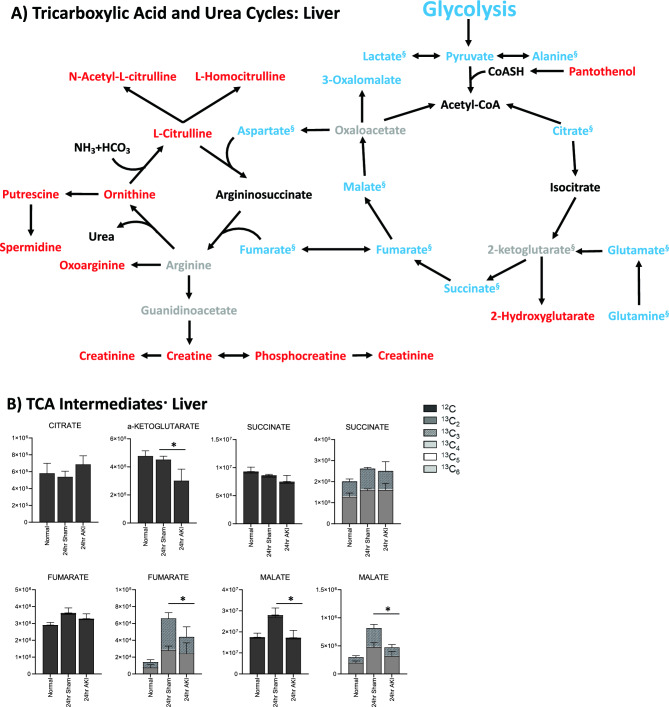

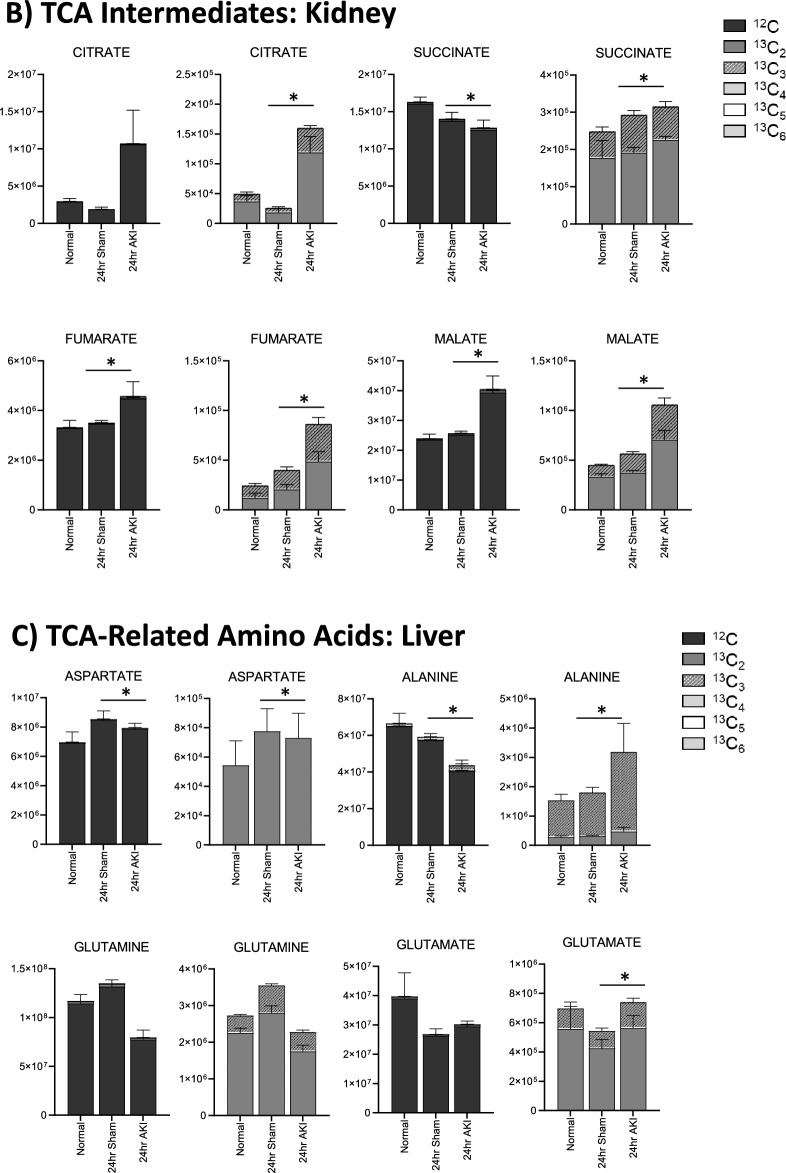

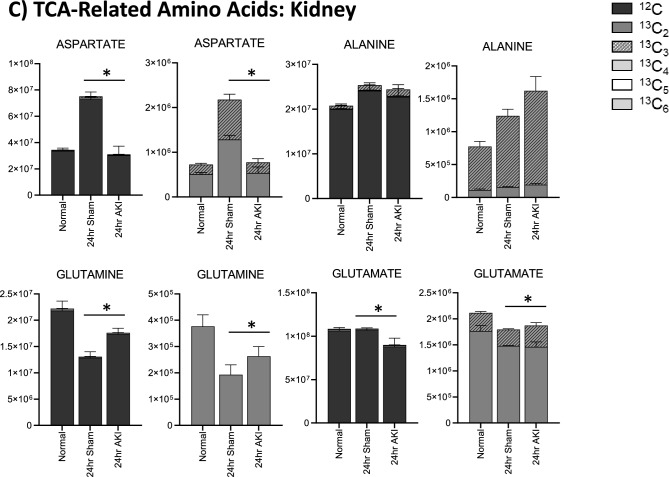
Pathway enrichment and flux analysis reveals that the TCA and urea cycles in the liver and kidney are impacted 24 h after AKI. (**A**) Pathway diagram including tricarboxylic acid (TCA) and urea cycles, illustrating steady state and labeled carbon study results in liver. Metabolites higher (red) or lower (blue) in AKI versus Sham mouse livers at 24 h post-procedure. Analytes in gray were detectable but not significantly different in AKI versus Sham. Analytes in black were not detectable through metabolomic analysis. Differences found in labeled carbon studies are designated by §. (**B**) Labeled carbon study results for TCA Intermediates in liver and kidney. (**C**) Labeled carbon study results for TCA-related Amino Acids in liver and kidney. For (**B**) and (**C**), *y*-axes represent peak areas (arbitrary units). For both liver and kidney, *n* = 8 Sham, *n* = 8 AKI. *P*-value < 0.05 (*) in Sham vs AKI. Data can be found in Supplementary Tables S6 and S7, respectively.

**Figure 6:**
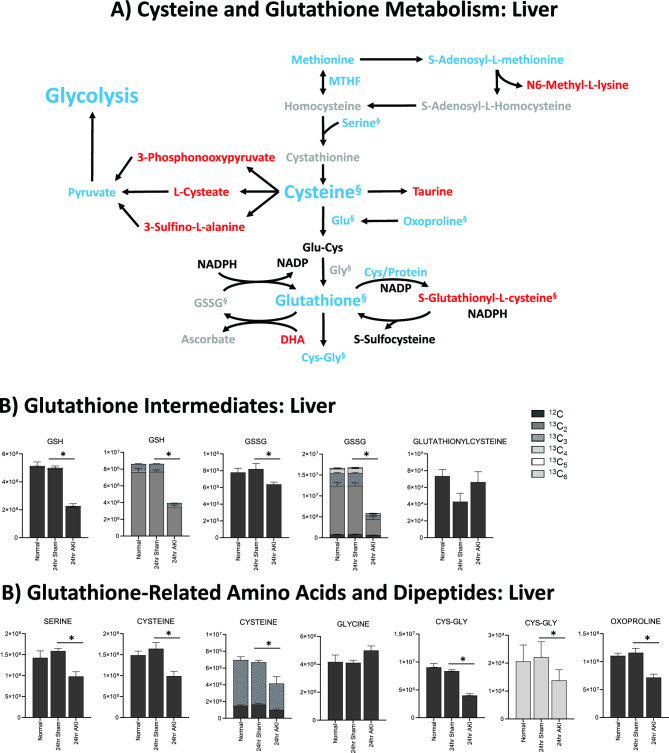

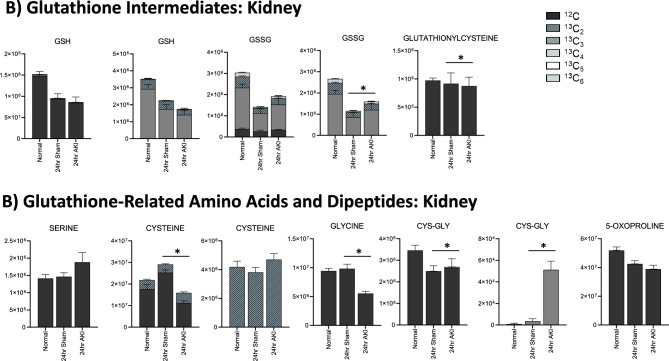
Glutathione is depleted in the liver and kidney 24 h after AKI, but the kidney has more glutathione intermediates available than the liver. (**A**) Pathway diagram including Cysteine and Glutathione Metabolism, illustrating steady state and labeled carbon study results in liver. Metabolites higher (red) or lower (blue) in AKI versus Sham mouse livers at 24 h post-procedure. Analytes in gray were detectable but not significantly different in AKI versus Sham. Analytes in black were not detectable through metabolomic analysis. Differences found in labeled carbon studies are designated by §. (**B**) Labeled carbon study results for Glutathione Intermediates, including Amino Acids and Dipeptides in liver and kidney. *y*-axes represent peak areas (arbitrary units). For both liver and kidney, *n* = 8 Sham, *n* = 8 AKI. *P*-value < 0.05 (*) in Sham vs AKI. Data can be found in Supplementary Tables S6 and S7, respectively.

Next, pathway enrichment analysis was performed using analytes that were significantly different at the 24-h time point. Analysis revealed enrichment for three major pathway groups (1) energy metabolism, (2) the urea cycle, and (3) cysteine and glutathione metabolism (Figs. 4A, 5A and 6A, respectively). Analytes and pathways of greatest significance, as well as p-value, FDR, and fold change for liver analytes measured at 24 h, can be found in Supplementary Tables S1 and S3, respectively, and are described in detail below.

#### Energy metabolism

The energy metabolism pathways affected include glycolysis, gluconeogenesis, the pentose phosphate pathway, and the tricarboxylic acid (TCA) cycle. Energy metabolites were nearly all lower in AKI versus sham, as is illustrated in Figs. 4A and 5A. Some of the lowest fold changes (fc = AKI/sham) were observed in D-glucose 6-phosphate (fc = 0.12), fructose 1,6-bisphosphate (fc = 0.21), and D-glyceraldehyde 3-phosphate (fc = 0.22), proximal key intermediates of glycolysis (Supplementary Table S3).

Labeled carbon studies (Figs. 4B,C and 5B,C, Supplementary Table S6) indicate lower total levels and increased intracellular uptake of labeled glucose in AKI versus sham (Fig. 4B). Flux through glycolysis is higher in AKI, as reflected by lower labeled phosphoglycerate, a glycolysis intermediate, and lower amounts of total with higher concentrations of labeled (or metabolized) alanine and lactate, glycolysis end-products (Fig. 4B). There were lower steady state levels of total analyte and higher flux (reflected by lower labeled species) through the pentose phosphate pathway (Fig. 4C). Malate, fumarate, succinate, and alpha-ketoglutarate in the TCA cycle were all lower in AKI versus sham (Fig. 5B), with lower levels of labeled species indicating increased flux through the TCA cycle, and lower overall intermediate availability. TCA-associated amino acids including aspartate, glutamine, glutamate, and alanine were generally found to be in lower concentration in AKI (Fig. 5A and C). Labeled carbon studies demonstrated variability in higher versus lower concentrations of labeled species in these amino acids, likely reflecting the complexity and dynamics of amino acid metabolism in multiple interacting pathways. Mean relative levels of liver analytes in sham and AKI cohorts, fold change, and p-values are found in the Supplementary Table S6. These data, taken together with the significant reduction in liver ATP levels observed at 24 h post-AKI (Fig. 1F), and the unchanged rate of oxidative phosphorylation (Fig. 2E), suggests greater consumption of ATP than the liver can generate.

#### The urea cycle

Urea cycle intermediates, including intermediates in arginine/ornithine metabolism such as putrescine, spermidine, creatinine, creatine, and phosphocreatine were uniformly higher in AKI versus sham in the liver at 24 h (Fig. 5A). Remarkably, the greatest fold change was seen in phosphocreatine (fc = 58.7), followed by citrulline (fc = 11.7), creatinine (fc = 6.8), *N*-acetyl-L-citrulline (fc = 6.4), creatine (fc = 6.2), and L-homocitrulline (fc = 6.1) (Supplementary Table S3). These data are consistent with the significantly increased levels of urea observed 24 h post-AKI (Fig. 1B), and likely indicate increased amino acid catabolism and resultant net positive nitrogen balance. They may also be indicative of tissue damage, though this is unlikely as whole cell damage was not seen histologically in liver.

### Glutathione metabolism

Glutathione-related metabolites were generally decreased in AKI versus sham (Fig. 6A). Glutathione (GSH) was significantly decreased in AKI, both at steady state and in the tracing studies, consistent with the significantly lower level of total glutathione observed 24 h post AKI (Fig. 1E). ^13^C*-*labeled GSH (Fig. 6B) was also lower in the liver in AKI versus sham, indicating decreased production, increased consumption, or increased export out of the liver. Cysteine, a direct and crucial component of glutathione synthesis, and cysteine/glutathione related analytes 5,10-methylene tetrahydrofolate (MTHF), *S*-adenosylmethionine (SAM), serine, and oxoproline were all lower in AKI (Fig. 6A). Labeled species were also lower in AKI (Fig. 6B), suggesting generally reduced production of these precursor analytes. Products of cysteine metabolism including taurine, 3-phosphonooxypyruvate, cysteic acid (cysteate), and 3-sulfino-L-alanine were higher in AKI indicating increased cysteine consumption, separate from glutathione synthesis (Fig. 6A). Interestingly, glutathionyl-cysteine was also higher in AKI, suggesting increased GSH consumption by glutathionylation (Fig. 6A,B).

In summary, metabolomic pathway analysis and labeled carbon studies demonstrate that, 24 h after AKI, the liver is characterized by energy deficiency, glutathione deficiency, glutathione precursor deficiency, and upregulated urea cycle metabolism. Energy deficiency is accompanied by reductions in and apparent increased consumption/metabolism of glycolytic, TCA, and PPP intermediates. Lower glutathione levels are accompanied by reduced availability of precursors (primarily cysteine) and increased downstream metabolites of glutathione precursors (primarily cysteine-related, non-glutathione metabolites).

### Kidney metabolomic analysis at 24 h demonstrates similarities to impacted pathways, but differences in intermediate availability, compared to the liver

**Figure 7:**
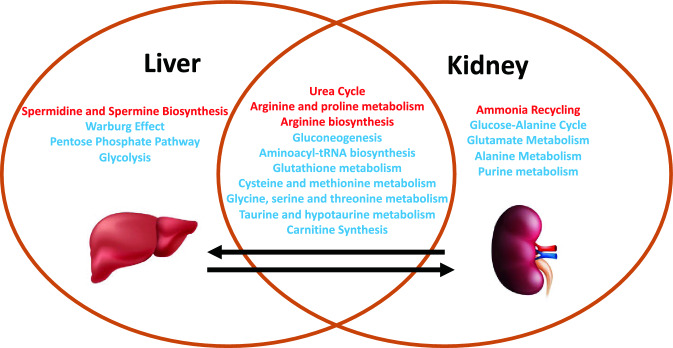
Comparison of affected metabolic pathways in the liver versus kidney 24 h after AKI. Venn diagram demonstrating similarities and differences in pathway enrichment in liver and kidney. Generally, enriched pathways demonstrated higher (red) or lower (blue) metabolite concentrations in AKI versus Sham at 24 h post-procedure.

Based on findings in liver metabolites at 24 h, we analyzed kidney tissue metabolites at 24 h in AKI versus sham mice^1^. In total, there were 90 of 177 detected metabolites (51%) that met significance by p-value and FDR (Supplementary Table S4). Pathway enrichment analysis in the kidney is compared with liver pathway enrichment in Fig. 7. Kidney pathway analysis demonstrated enrichment for glutathione and cysteine related metabolism and urea cycle and arginine related metabolism, much like those found in the liver (Supplementary Table S5). Unlike the liver, energy related pathways including glycolysis, PPP, and the TCA cycle were not enriched. Generally, analytes in the glutathione and cysteine pathways were lower in AKI versus sham (notably cysteine, gamma-glutamyl-cysteine, homocysteine, *S*-adenosylhomocysteine (SAH), and methionine). Total glutathione was not significantly different between AKI and sham^1^. As in liver, those analytes related to nitrogen balance, arginine, and the urea cycle (notably citrulline, homocitrulline, *N*-acetylcitrulline, guanidinosuccinic acid, putrescine, creatine, creatinine, phosphocreatine, 2-oxoarginine, guanidinoacetate, arginine, ornithine, and carglumic acid) were higher in AKI compared with sham.

### Kidney labeled carbon analysis at 24 h supports greater pools of intermediates in AKI compared with the liver

In labeled carbon analyses, we compared kidney metabolites to liver metabolites. In glycolysis (Fig. 4B), generally, labeled carbon species including hexose phosphate, bisphosphoglycerate, lactate, and alanine were higher in AKI versus sham, indicating increased flux through the glycolytic pathway in both liver and kidney. Interestingly, in the kidney, total concentrations of analytes (including light, unlabeled species) were higher in AKI than sham, whereas in the liver they were lower in AKI. This reflects a greater abundance of energy resources available in the kidney. Analytes from the pentose phosphate pathway (Fig. 4C) demonstrated lower total and labeled species in AKI compared to sham in both the liver and kidney.

TCA cycle intermediates were generally higher (both total and labeled) in the AKI versus sham kidney (Fig. 5B), particularly fumarate and malate. This is opposite the pattern seen in the liver, and again suggests that there are more abundant energy intermediates in the AKI kidney compared to the liver. TCA-related amino acids (Fig. 5C) were variable in both tissues, with no clear unifying pattern. Mean relative levels of kidney analytes in sham and AKI cohorts, fold change, and *p*-values are found in the Supplementary Table S7.

Finally, in the liver, total and oxidized glutathione were both lower in AKI versus sham; in the kidney, however, total glutathione is similar in AKI versus sham, and oxidized glutathione is higher in AKI compared to sham (Fig. 6B). This suggests that, whereas in the AKI liver glutathione is generally depleted, in the AKI kidney there is no loss of glutathione, and glutathione is being appropriately oxidized in the setting of stress. This is consistent with pathway and general metabolomic analyses. In the kidney, glutathione precursors are higher in AKI versus sham, whereas in the AKI liver they are lower. This, again, suggests that crucial resources in both energy metabolism and oxidative stress mitigation are more available in the AKI kidney compared to the AKI liver.

## Discussion

Herein, we performed metabolic assessment and glucose tracing studies after ischemic AKI in mice and found that small molecule metabolism in the liver is substantially affected after AKI, which reveal numerous novel findings, summarized as follows:

1. Depletion of glycolysis and TCA cycle intermediates suggests increased flux through glycolysis and the TCA cycle in both kidney and liver.
2. Depletion of hepatic glutathione and glutathione intermediates (cysteine, glycine, glutamine) in the absence of increased hepatic ROS suggests increased hepatic glutathione consumption or export relative to production suggesting excess systemic oxidative stress.
3. Depletion of hepatic ATP despite unchanged rate of mitochondrial respiration indicative of increased ATP consumption relative to production.
4. Increased hepatic and renal urea cycle intermediates and related compounds (citrulline, citrulline derivatives, ornithine, creatine, phosphocreatine, creatinine, and polyamines) suggest upregulation of the urea cycle and hypercatabolism.
5. Importantly, all these effects occurred in the absence of demonstrable histologic liver injury, which has been suggested to be the cause of liver derangements in other murine studies of AKI.

Thus, the findings in this report bring to light novel characteristics of AKI-related kidney-liver cross talk and lend additional insight into the complex systemic nature of AKI in general.

Previous studies have demonstrated that AKI induces liver injury which is characterized by upregulation of proinflammatory cytokines and chemokines^29–31^, neutrophil infiltration, and hepatic cell necrosis^30,32^. Effects on redox balance have also been observed including reduced levels of glutathione^31–33^, elevated levels of oxidized proteins and lipids, and reduced gene expression of enzymes and transcription factors associated with glutathione synthesis^33^. Mitochondrial function, ROS generation, and metabolomics analysis—as reported herein—have not previously been examined. An overarching paradigm suggested by previous studies has been that the systemic inflammation induced by AKI leads to hepatic inflammation, hepatic cell necrosis, excess ROS production, and glutathione consumption. In our model of AKI, neither hepatic neutrophil infiltration nor hepatic cell necrosis were observed. Furthermore, hepatic superoxide levels were not affected relative to sham and mitochondrial function was normal. Thus, our novel data indicate that AKI-induced liver injury is not necessary to cause the observed metabolic effects. Rather, our data suggest that the changes in energy and redox balance reflect kidney-liver cross talk in AKI, possibly reflecting the use of resources from the liver by the kidney to mitigate kidney cellular damage in AKI.

Our data demonstrate that multiple energy pathways were affected in the liver after AKI including glycolysis, gluconeogenesis, and the TCA cycle and were characterized by the depletion of multiple energy substrates and reduced levels of ATP. These changes in energy metabolism are consistent with the known effect of AKI as a hypercatabolic state and may reflect excess consumption of energy resources, particularly amino acids. While the mechanistic underpinnings behind this energy substrate depletion remain to be elucidated, these results are consistent with previously published data demonstrating reduced ATP and energy substrate depletion in the heart^6^ and lung^15^. Together, these data indicate that AKI has widespread effects on energy metabolism in multiple organs and may be characterized globally as an energy-deficient state.

In addition to the changes in hepatic energy pathways, our data demonstrate that AKI affects redox balance in the liver as judged by reduced levels of glutathione and its intermediates. The liver is the major source of glutathione production and exportation, playing an important role in regulating local and systemic redox balance. Hepatic glutathione levels have been shown to be depleted within 6 h of ischemic AKI^31,32^, which is consistent with our data demonstrating lower levels of hepatic glutathione at both 4 and 24 h after ischemic AKI. Furthermore, we also observed that substrates related to glutathione synthesis were dramatically reduced in the liver after AKI, including cysteine, glycine, and glutamine. The lack of these amino acid precursors in conjunction with metabolic flux analysis suggest decreased hepatic glutathione production relative to demand, as evidenced by the paucity of heavy-labeled isotope tracer present in glutathione. This is further supported by our prior published data describing decreased plasma and heart total glutathione levels 24 h after AKI^1^. Since ROS levels were not directly increased in the liver (as judged by mitochondrial superoxide levels) and mitochondrial function was normal, these data suggest that glutathione consumption is likely a consequence of systemic derangements in redox balance induced by kidney injury as opposed to a direct effect of inflammation or cell death within the liver.

In addition to changes in energy and glutathione metabolism, the third major pathway affected in the liver after AKI was urea metabolism. We found elevations in multiple analytes involved in the urea cycle and toxic nitrogen excretion in the liver including citrulline, citrulline derivatives, ornithine, creatine, phosphocreatine, creatinine, and polyamines. Urea, also known as BUN, is a small molecule that is freely filtered by the kidney and excreted into the urine. Thus, reduced kidney function (i.e., reduced glomerular filtration rate (GFR)) is associated with a rise in BUN as observed herein. Elevations in BUN—as well as creatinine—are used clinically to identify and track changes in kidney function, whether acute or chronic. Thus, the rise in BUN and creatinine noted in our AKI model is consistent with the fall in kidney function from AKI; in previous studies, we have demonstrated that the level of creatinine and BUN rise observed is associated with histologic kidney tubular injury and a > 90% loss of kidney function as judged by measured GFR^34^.

Independent of loss of kidney function, a rise in BUN may also occur as a result of hypercatabolism from the excess breakdown of amino acids. This hypercatabolism is evident by our data demonstrating depletion of amino acids and upregulation of the urea cycle as judged by elevated levels of urea cycle metabolites such as citrulline. Increased catabolism of amino acids leads to the release of the amine group from amino acids whose carbon skeletons are used to replete intermediates of energy metabolism. Increased amine release leads to the formation of ammonia. The primary function of the urea cycle is to process this excess ammonia into urea which can then be eliminated through excretion by the kidney. Ammonia—and its associated nitrogen—is a toxic byproduct that requires activation of the urea cycle for homeostasis and prevention of ammonia toxicity. Since kidney function is reduced during AKI, activation of the urea cycle and subsequent urea generation together lead to a substantial rise in BUN^14,35^.

We examined the effect of AKI on the kidney and compared the effects within the kidney and liver together. Normally, the kidney and liver rely heavily on oxidative phosphorylation for energy production as both the liver and the kidney are dense with mitochondria. The metabolic effects of AKI within the kidney are well-described: disruption of mitochondrial structure, dynamics, biogenesis, and basic electron transport chain function are all present^36^; lipid metabolism is reduced and NAD^+^ generation is impaired^37^; and gluconeogenesis is suppressed^38^ in part due to depletion of pyruvate and lactate^39^. In the kidney, we observed widespread disruption in energy metabolism and multiple molecules involved in glycolysis, gluconeogenesis, and the TCA cycle were depleted, indicating a more global disruption in energy metabolism than has been previously appreciated. Compared to the liver, we observed that the kidney has: (1) similar increased use of energy-related metabolites and production of urea cycle and ammonia-related intermediates, suggesting a shared catabolism in AKI, and (2) higher pools of energy metabolism intermediates in AKI. In contrast to the kidney, the liver has relatively less “reserve” pools of analytes to recover flux through these pathways, and thus energy production.

Together, our data suggest that there is significant cross talk between the injured kidney and the liver during ischemic AKI. Our data are consistent with the notion that energy substrates and glutathione produced in the liver are shuttled systemically and to the kidney to meet the metabolic demands of kidney repair and maintenance of redox balance. In light of these data, we suggest therapies that target energy metabolism and ROS generation to improve kidney function recovery may need to also target the energy and redox derangements observed in the liver. This is especially true regarding nutritional supplementation in AKI. Current nutrition recommendations advise that patients with AKI receive additional protein supplementation to address the hypercatabolic state. Since our data suggest that the urea cycle in AKI is already overloaded, it is uncertain what levels of protein intake are necessary to meet metabolic needs and at what point a high protein intake will overwhelm the urea cycle and ammoniagenesis. Perhaps a diet with increased glucose (but not fructose) would better suit the needs of the AKI patient given the metabolism observed here.

In conclusion, we demonstrate that widespread changes in energy metabolism and redox balance are evident in the liver after AKI. Overall, these data suggest that AKI is systemically characterized by energy substrate depletion and excess oxidative stress. Strategies to address kidney function recovery to improve metabolism or oxidative stress must also address these within the liver.

### Supplementary Information

Supplementary Information.

## Data Availability

The datasets generated during and/or analyzed during the current study are available from the corresponding author on reasonable request.

## References

[1] Uchino S, Bellomo R, Goldsmith D, Bates S, Ronco C (2006). An assessment of the RIFLE criteria for acute renal failure in hospitalized patients. Crit. Care Med..

[2] Faubel S, Shah PB (2016). Immediate consequences of acute kidney injury: The impact of traditional and nontraditional complications on mortality in acute kidney injury. Adv. Chronic Kidney Dis..

[3] Hassoun HT (2007). Ischemic acute kidney injury induces a distant organ functional and genomic response distinguishable from bilateral nephrectomy. Am. J. Physiol. Renal. Physiol..

[4] Kramer AA (1999). Renal ischemia/reperfusion leads to macrophage-mediated increase in pulmonary vascular permeability. Kidney Int..

[5] Kelly KJ (2003). Distant effects of experimental renal ischemia/reperfusion injury. J. Am. Soc. Nephrol..

[6] Fox BM (2019). Metabolomics assessment reveals oxidative stress and altered energy production in the heart after ischemic acute kidney injury in mice. Kidney Int..

[7] Kim M, Park SW, D'Agati VD, Lee HT (2011). Isoflurane activates intestinal sphingosine kinase to protect against bilateral nephrectomy-induced liver and intestine dysfunction. Am. J. Physiol. Renal. Physiol..

[8] Yildirim A, Gumus M, Dalga S, Sahin YN, Akcay F (2003). Dehydroepiandrosterone improves hepatic antioxidant systems after renal ischemia-reperfusion injury in rabbits. Ann. Clin. Lab. Sci.

[9] Liu M (2008). Acute kidney injury leads to inflammation and functional changes in the brain. J. Am. Soc. Nephrol..

[10] Park SW (2012). Paneth cell-mediated multiorgan dysfunction after acute kidney injury. J. Immunol..

[11] Awad AS, Okusa MD (2007). Distant organ injury following acute kidney injury. Am. J. Physiol. Renal. Physiol..

[12] Van Biesen W, Lameire N, Vanholder R, Mehta R (2007). Relation between acute kidney injury and multiple-organ failure: the chicken and the egg question. Crit. Care Med..

[13] Berbel MN, Goes CR, Balbi AL, Ponce D (2014). Nutritional parameters are associated with mortality in acute kidney injury. Clinics (Sao Paulo)..

[14] Schrier RW (1979). Nephrology forum: Acute renal failure. Kidney Int..

[15] Ambruso SL (2021). Lung metabolomics after ischemic acute kidney injury reveals increased oxidative stress, altered energy production, and ATP depletion. Am. J. Physiol. Lung Cell Mol. Physiol..

[16] Hepokoski M (2021). Altered lung metabolism and mitochondrial DAMPs in lung injury due to acute kidney injury. Am. J. Physiol. Lung Cell Mol. Physiol..

[17] Klein CL (2008). Interleukin-6 mediates lung injury following ischemic acute kidney injury or bilateral nephrectomy. Kidney Int..

[18] D'Alessandro A (2015). Early hemorrhage triggers metabolic responses that build up during prolonged shock. Am. J. Physiol. Regul. Integr. Comp. Physiol..

[19] Nemkov T, Reisz JA, Gehrke S, Hansen KC, D'Alessandro A (2019). High-throughput metabolomics: Isocratic and gradient mass spectrometry-based methods. Methods Mol. Biol..

[20] Kanehisa M, Goto S (2000). KEGG: Kyoto encyclopedia of genes and genomes. Nucleic Acids Res..

[21] Kanehisa M (2019). Toward understanding the origin and evolution of cellular organisms. Protein Sci..

[22] Kanehisa M, Furumichi M, Sato Y, Kawashima M, Ishiguro-Watanabe M (2023). KEGG for taxonomy-based analysis of pathways and genomes. Nucleic Acids Res..

[23] Nemkov T, Hansen KC, D'Alessandro A (2017). A three-minute method for high-throughput quantitative metabolomics and quantitative tracing experiments of central carbon and nitrogen pathways. Rapid Commun. Mass Spectrom..

[24] Monks J (2018). Maternal obesity during lactation may protect offspring from high fat diet-induced metabolic dysfunction. Nutr. Diabetes..

[25] Lanaspa MA (2018). Ketohexokinase C blockade ameliorates fructose-induced metabolic dysfunction in fructose-sensitive mice. J. Clin. Invest..

[26] Hoke TS (2007). Acute renal failure after bilateral nephrectomy is associated with cytokine-mediated pulmonary injury. J. Am. Soc. Nephrol..

[27] Gnaiger EKA, Heldmaier GKM (2000). Mitochondria in the cold. Life in the Cold.

[28] Elajaili HB, Hernandez-Lagunas L, Ranguelova K, Dikalov S, Nozik-Grayck E (2019). Use of electron paramagnetic resonance in biological samples at ambient temperature and 77 K. J. Vis. Exp..

[29] Xia J, Wishart DS (2016). Using MetaboAnalyst 3.0 for comprehensive metabolomics data analysis. Curr. Protoc. Bioinformatics..

[30] Hochberg Y, Benjamini Y (1990). More powerful procedures for multiple significance testing. Stat. Med..

[31] Han SJ, Li H, Kim M, D'Agati V, Lee HT (2019). Intestinal Toll-like receptor 9 deficiency leads to Paneth cell hyperplasia and exacerbates kidney, intestine, and liver injury after ischemia/reperfusion injury. Kidney Int..

[32] Park SW (2011). Cytokines induce small intestine and liver injury after renal ischemia or nephrectomy. Lab Invest..

[33] Serteser M (2002). Changes in hepatic TNF-alpha levels, antioxidant status, and oxidation products after renal ischemia/reperfusion injury in mice. J. Surg. Res..

[34] Golab F (2009). Ischemic and non-ischemic acute kidney injury cause hepatic damage. Kidney Int..

[35] Shang Y, Siow YL, Isaak CK, Karmin O (2016). Downregulation of glutathione biosynthesis contributes to oxidative stress and liver dysfunction in acute kidney injury. Oxid. Med. Cell. Longev..

[36] Golab F (2009). Hepatic changes during various periods of reperfusion after induction of renal ischemia in rats. Transplant Proc..

[37] Skrypnyk NI (2020). IL-6-mediated hepatocyte production is the primary source of plasma and urine neutrophil gelatinase-associated lipocalin during acute kidney injury. Kidney Int..

[38] Parsons FM, Hobson SM, Blagg CR, McCracken BH (1961). Optimum time for dialysis in acute reversible renal failure. Description and value of an improved dialyser with large surface area. Lancet..

[39] Clark AJ, Parikh SM (2020). Mitochondrial metabolism in acute kidney injury. Semin. Nephrol..

