# Supplementary material for: Disruption in glutathione metabolism and altered energy production in the liver and kidney after ischemic acute kidney injury in mice
Source: Sci Rep. 2024 Jun 15;14:13862. doi: 10.1038/s41598-024-64586-4 (PMC11180093; doi:10.1038/s41598-024-64586-4)
Supplement: Supplementary file 1 — Supplementary Information. [file 41598_2024_64586_MOESM1_ESM.pdf]

Supplementary Figure S1. Liver H&E histology 24 hours after AKI. Slight reactive changes such as increased intracellular lipid vesicles in hepatocytes (arrows) were noted in two of the eight liver tissue H&E sections 24 hours after AKI. These reactive changes may be associated with liver “stress,” but are not indicative of cell injury, cell death, or inflammation.

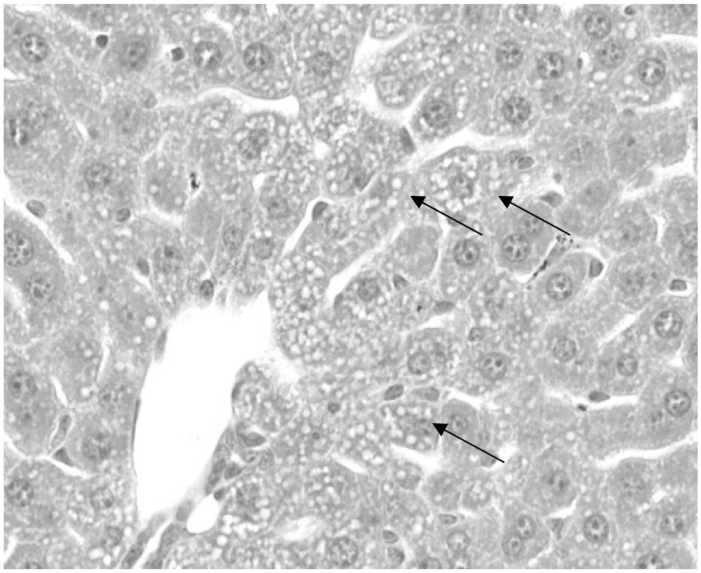

AKI 24hr

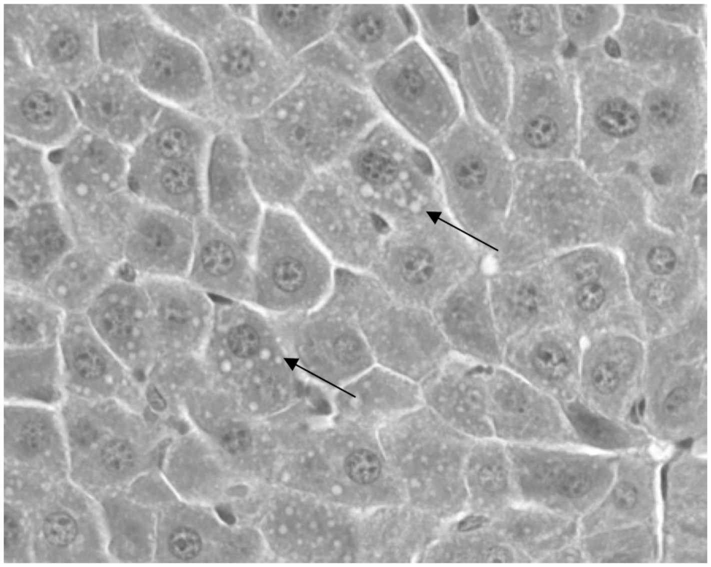

AKI 24hr

Supplementary Figure S2. Principal component analysis (PCA) plot of all liver samples from untargeted metabolomics. Principal component analysis for steady state metabolomics of liver specimens from normal, sham, and AKI at 4 hours, 24 hours, and 7 days after surgery. Data was sum-normalized and autoscaled using MetaboAnalyst 5.0. QC = quality control (i.e., technical replicate injections). Blank = metabolite extraction buffer.

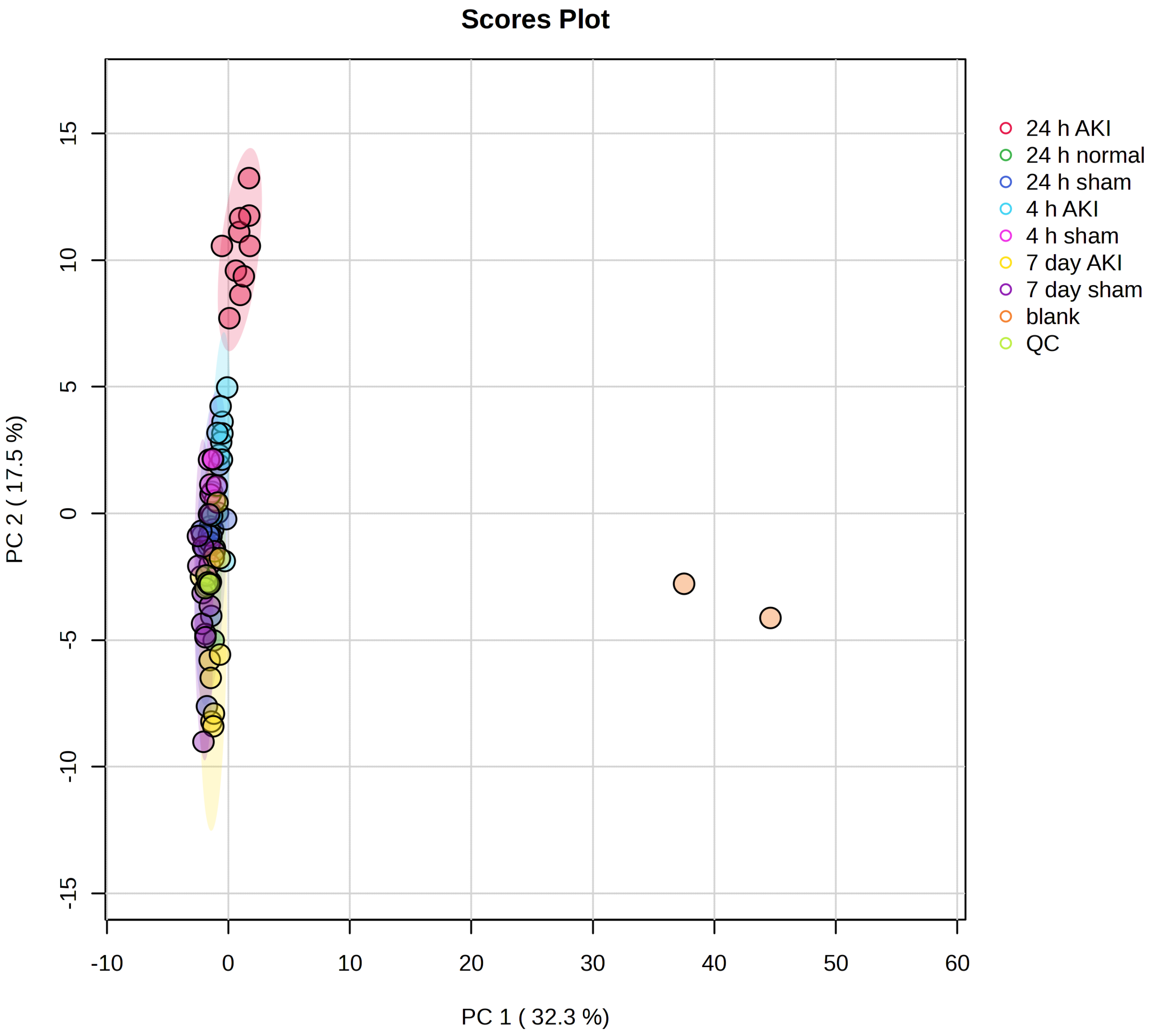

Supplementary Table S1. Untargeted metabolomics analysis of the liver at 4 hours, 24 hours, and 7 days after AKI. 100 of 141 annotated metabolites that were identified by UHPLC-MS were significantly different amongst the three experimental groups by univariate ANOVA without *post hoc* testing. Statistical analyses were performed using MetaboAnalyst 3.0.

Compound Name and Identification Number			ANOVA All Timepoints			4-hour		24-hour		7-day	
**KEGG ID**	**Compound Name**	**MetaboAnalyst Compatible Name**	**F-Value**	**P-Value**	**BH FDR**	**FC (AKI/Sham)**	**p-value**	**FC (AKI/Sham)**	**p-value**	**FC (AKI/Sham)**	**p-value**
C02354	2',3'-Cyclic CMP	2',3'-Cyclic CMP	42.45	1.89E-19	2.66E-17	1.45	1.50E-03	13.37	8.39E-09	1.40	0.249
C02305	Phosphocreatine	Phosphocreatine	36.53	5.89E-18	4.15E-16	1.06	0.402	53.68	1.89E-06	2.01	0.987
C13085	Sodium glucuronate	Glucuronate	27.99	1.96E-15	9.23E-14	0.83	0.048	0.20	4.13E-10	0.87	0.057
C06381	Methylenediurea	NA	26.17	7.99E-15	2.82E-13	1.28	0.621	6.03	4.55E-06	2.47	0.084
C00300	Creatine	Creatine	24.60	2.84E-14	7.27E-13	1.22	0.687	6.17	6.14E-06	1.93	0.111
C00327	L-Citrulline	L-Citrulline	24.49	3.09E-14	7.27E-13	1.70	0.030	11.71	1.95E-06	2.98	0.014
C00346	Ethanolamine phosphate	Ethanolamine phosphate	22.76	1.35E-13	2.71E-12	1.09	0.306	2.06	3.13E-04	1.02	0.789
C02350	(S)(+)-Allantoin	(S)(+)-Allantoin	22.57	1.59E-13	2.80E-12	1.81	1.02E-03	3.15	7.75E-06	1.16	0.013
C00864	Pantothenate	Pantothenate	20.94	6.83E-13	1.07E-11	2.55	5.09E-04	3.89	9.64E-05	1.00	0.635
C01068	D-Ribitol 5-phosphate	D-Ribitol 5-phosphate	18.59	6.45E-12	8.99E-11	0.85	0.056	0.44	1.23E-08	0.91	0.059
C00121	D-Ribose	D-Ribose	18.42	7.65E-12	8.99E-11	1.79	6.51E-03	4.49	2.20E-05	3.13	1.27E-03
C00259	L-Arabinose	L-Arabinose	18.42	7.65E-12	8.99E-11	1.79	6.51E-03	4.49	2.29E-05	3.13	1.27E-03
C00906	5-6-Dihydrothymine	Dihydrothymine	17.18	2.69E-11	2.77E-10	1.17	0.903	4.03	1.32E-07	2.77	7.81E-03
C03410	N-Glycoloyl-neuraminate	N-Glycoloyl-neuraminate	17.16	2.75E-11	2.77E-10	1.02	0.987	1.54	0.050	1.01	0.606
C15532	N-Acetyl-L-citrulline	N-Acetyl-L-citrulline	16.84	3.87E-11	3.64E-10	0.81	0.033	6.37	3.03E-04	1.68	0.103
C06156	alpha-D-Glucosamine 1-phosphate	alpha-D-Glucosamine 1-phosphate	14.30	6.35E-10	5.60E-09	0.80	0.036	0.47	2.31E-06	0.94	0.242
C02965	D-Glucose 6-phosphate	D-Glucose 6-phosphate	14.02	8.83E-10	7.32E-09	0.79	0.113	0.12	1.22E-06	1.09	0.795
C01236	D-Glucono-1-5-lactone 6-phosphate	6-Phosphonoglucono-D-lactone	13.34	1.97E-09	1.54E-08	0.78	0.077	0.30	6.46E-04	1.06	0.854
C03792	N-Acyl-D-mannosaminolactone	NA	13.16	2.45E-09	1.82E-08	0.83	0.314	0.10	5.44E-06	0.65	0.116
C04287	3D-(3-5/4)-Trihydroxycyclohexane-1-2-dione	3D-(3,5/4)-Trihydroxycyclohexane-1,2-dione	12.81	3.74E-09	2.64E-08	0.83	0.032	0.28	2.58E-07	1.23	0.436
C02427	L-Homocitrulline	L-Homocitrulline	12.61	4.82E-09	3.23E-08	1.23	0.072	6.09	1.35E-05	3.35	0.018
C00791	Creatinine	Creatinine	12.42	6.09E-09	3.91E-08	2.32	3.02E-04	6.80	5.10E-03	3.43	3.63E-03
C00270	N-Acetylneuraminate	N-Acetylneuraminate	12.25	7.49E-09	4.59E-08	1.00	0.450	1.40	0.014	1.40	0.632
C03771	5-Guanidino-2-oxopentanoate	5-Guanidino-2-oxopentanoate	12.18	8.16E-09	4.80E-08	2.63	6.67E-03	5.62	4.68E-05	1.16	0.412
C00568	4-Aminobenzoate	4-Aminobenzoate	10.62	6.20E-08	3.50E-07	0.87	0.741	0.72	0.158	2.35	5.11E-03
C00499	Allantoate	Allantoate	10.18	1.13E-07	6.14E-07	1.25	0.064	2.88	6.91E-04	0.83	0.735
C00119	5-Phospho-alpha-D-ribose 1-diphosphate	5-Phospho-alpha-D-ribose 1-diphosphate	9.63	2.44E-07	1.27E-06	1.16	0.070	0.28	5.98E-06	0.59	0.065
C00149	Malate	Malate	9.45	3.16E-07	1.59E-06	1.02	0.120	0.35	3.75E-06	1.23	0.873
C16618	6-Thioxanthine 5--monophosphate	6-Thioxanthine 5'-monophosphate	9.32	3.76E-07	1.83E-06	1.65	0.085	3.18	9.30E-04	0.64	0.886
C01602	Ornithine	Ornithine	9.23	4.27E-07	2.01E-06	0.94	0.831	2.63	8.69E-04	1.16	0.291
C00199	Pentose phosphates (isobars)	D-Ribulose 5-phosphate	9.10	5.16E-07	2.35E-06	1.02	0.409	0.80	0.049	1.17	0.316
C00134	Putrescine	Putrescine	8.79	8.06E-07	3.55E-06	1.25	0.132	2.35	2.11E-03	1.38	8.82E-04
C11745	Succinyl sulfathiazole	NA	8.52	1.20E-06	5.13E-06	0.92	0.705	1.66	1.38E-03	2.49	0.014
C00183	Valine	Valine	8.25	1.79E-06	7.43E-06	1.14	0.918	0.79	0.659	1.34	0.746
C02728	N6-Methyl-L-lysine	N6-Methyl-L-lysine	8.22	1.89E-06	7.61E-06	0.96	0.212	1.71	4.44E-03	3.49	1.98E-03
C00345	6-Phospho-D-gluconate	6-Phospho-D-gluconate	7.93	2.93E-06	1.15E-05	0.46	2.99E-03	0.19	1.07E-05	1.55	0.760
C00019	S-Adenosyl-L-methionine	S-Adenosyl-L-methionine	7.57	5.11E-06	1.95E-05	0.87	0.106	0.51	2.32E-03	1.28	0.206
C05422	Dehydroascorbate	Dehydroascorbate	7.48	5.86E-06	2.17E-05	1.25	0.680	1.34	1.60E-03	1.31	0.035
C03232	3-Phosphonooxypyruvate	3-Phosphonooxypyruvate	7.43	6.29E-06	2.28E-05	1.08	0.091	1.93	1.58E-08	0.52	0.711
C03758	Dopamine	Dopamine	7.05	1.15E-05	4.06E-05	0.74	0.042	0.88	0.061	0.81	0.781
C02589	S-Acylglutathione	S-Lactoylglutathione	7.02	1.19E-05	4.11E-05	1.26	0.911	0.91	0.282	1.58	0.146
C00047	Lysine	Lysine	6.82	1.66E-05	5.57E-05	1.07	0.837	1.04	0.997	1.83	0.058
C00245	Taurine	Taurine	6.27	4.05E-05	1.33E-04	1.13	0.317	2.35	3.15E-05	2.30	0.029
C01990	3-Oxalomalate	3-Oxalomalate	6.23	4.27E-05	1.37E-04	0.76	0.063	0.46	3.75E-05	1.06	0.336
C00631	2/3-Phospho-D-glycerate	2-Phospho-D-glyceric acid	6.08	5.54E-05	1.74E-04	1.35	0.423	0.56	4.06E-04	1.04	0.331
C00118	D-Glyceraldehyde 3-phosphate/Glycerone phosphate	D-Glyceraldehyde 3-phosphate	6.05	5.83E-05	1.79E-04	1.29	0.677	0.22	1.93E-04	0.92	0.954
C03339	2-3-Bisphosphoglycerate	2,3-Diphosphoglyceric acid	5.89	7.62E-05	2.27E-04	0.79	0.887	0.45	2.83E-03	0.13	0.033

C00022	Pyruvate	Pyruvate	5.88	7.74E-05	2.27E-04	0.93	0.068	0.76	2.75E-03	1.09	0.845
C00051	Glutathione	Glutathione	5.50	1.47E-04	4.23E-04	0.55	0.019	0.53	2.21E-03	1.02	0.856
C00049	Aspartate	Aspartate	5.48	1.50E-04	4.23E-04	0.87	0.083	0.56	3.77E-03	0.87	0.619
C00093	Glycerol 3-phosphate	Glycerol 3-phosphate	5.47	1.54E-04	4.27E-04	1.06	0.298	0.28	1.79E-04	1.69	0.194
C00294	Inosine	Inosine	5.38	1.80E-04	4.89E-04	0.92	0.318	0.83	0.146	1.21	0.854
C01233	sn-glycero-3-Phosphoethanolamine	1-Acyl-sn-glycero-3-phosphoethanolamine	5.29	2.08E-04	5.53E-04	0.87	0.080	0.64	1.02E-03	0.91	0.209
C01157	trans-4-Hydroxy-L-proline	trans-4-Hydroxy-L-proline	5.27	2.15E-04	5.61E-04	0.90	0.244	0.73	0.070	2.11	0.119
C00008	ADP	ADP	5.22	2.36E-04	6.06E-04	0.91	0.772	1.23	0.648	1.02	0.938
C00097	Cysteine	Cysteine	5.18	2.52E-04	6.28E-04	0.72	0.082	0.66	4.98E-03	1.01	0.525
C00315	Spermidine	Spermidine	5.18	2.54E-04	6.28E-04	0.81	0.692	1.97	3.65E-05	0.83	0.568
C00534	Pyridoxamine	Pyridoxamine	5.14	2.69E-04	6.48E-04	1.11	0.242	1.04	0.243	1.34	0.125
C06738	cis-p-Coumarate	cis-p-Coumarate	5.14	2.71E-04	6.48E-04	0.97	0.217	0.59	9.01E-04	1.42	0.632
C00073	Methionine	Methionine	4.95	3.74E-04	8.80E-04	0.68	0.040	0.68	0.020	1.61	0.535
C00956	L-2-Aminoadipate	L-2-Aminoadipate	4.90	4.12E-04	9.52E-04	0.62	0.067	1.41	0.158	0.65	0.474
C01419	Cys-Gly	Cys-Gly	4.72	5.60E-04	1.27E-03	0.60	0.025	0.53	2.11E-03	1.00	0.906
C03153	N5-Methyl-L-glutamine	N-methyl-L-glutamic Acid	4.68	6.01E-04	1.34E-03	0.83	0.076	0.68	3.17E-03	1.86	0.125
C06222	Sedoheptulose 1-phosphate	D-Sedoheptulose 7-phosphate	4.64	6.49E-04	1.43E-03	0.80	0.038	0.45	0.032	1.45	0.689
C10906	D-Fructose	D-Fructose	4.53	7.83E-04	1.67E-03	0.35	0.016	0.24	1.51E-03	1.88	0.572
C00082	Tyrosine	Tyrosine	4.53	7.92E-04	1.67E-03	0.93	0.122	0.56	3.26E-03	1.50	0.513
C02630	2-Hydroxyglutarate/Citramalate	2-Hydroxyglutarate	4.52	7.95E-04	1.67E-03	0.95	0.075	1.65	1.24E-03	1.19	0.550
C03139	N-Amidino-L-aspartate	N-Amidino-L-aspartate	4.38	1.02E-03	2.12E-03	1.22	0.762	0.85	0.631	1.97	0.252
C00519	Hypotaurine	Hypotaurine	4.33	1.12E-03	2.29E-03	0.84	0.201	0.91	0.589	0.44	0.072
C00079	Phenylalanine	Phenylalanine	4.21	1.39E-03	2.81E-03	0.98	0.243	0.89	0.130	1.60	0.199
C00065	Serine	Serine	4.16	1.52E-03	3.01E-03	0.84	0.156	0.60	3.19E-04	1.06	0.867
C00152	Asparagine	Asparagine	4.01	1.98E-03	3.89E-03	0.81	0.170	0.89	0.305	1.35	0.609
C00042	Succinate	Succinate	4.00	2.02E-03	3.90E-03	0.69	0.092	0.55	0.030	1.11	0.465
C15699	gamma-L-Glutamylputrescine	gamma-L-Glutamylputrescine	3.96	2.21E-03	4.20E-03	1.03	0.404	0.66	0.061	1.14	0.981
C00013	Diphosphate	Diphosphate	3.93	2.31E-03	4.35E-03	0.99	0.424	1.15	0.023	0.92	0.146
C01432	Lactate	Lactate	3.85	2.66E-03	4.93E-03	0.86	0.058	0.49	1.46E-03	1.95	0.567
C00037	Glycine	Glycine	3.79	2.96E-03	5.43E-03	0.98	0.218	1.06	0.364	1.28	0.205
C00437	N-Acetylornithine	N-Acetylornithine	3.78	3.06E-03	5.52E-03	0.95	0.112	1.23	0.401	1.49	0.490
C01026	Dimethylglycine	Dimethylglycine	3.73	3.34E-03	5.96E-03	0.96	0.100	1.37	0.200	1.23	0.993
C02930	2-Methyleneglutarate	2-Methyleneglutarate	3.70	3.54E-03	6.24E-03	0.85	0.073	0.55	8.89E-04	1.51	0.865
C17220	2-Oxo-7-methylthioheptanoic acid	2-Oxo-7-methylthioheptanoic acid	3.57	4.42E-03	7.69E-03	1.20	0.027	1.20	0.076	1.08	0.423
C00105	UMP	UMP	3.46	5.47E-03	9.41E-03	1.00	0.478	1.02	0.612	0.82	0.168
C05526	S-Glutathionyl-L-cysteine	S-Glutathionyl-L-cysteine	3.43	5.73E-03	9.73E-03	0.99	0.885	2.16	1.12E-03	0.80	0.883
C00031	D-Glucose	Alpha-D-Glucose	3.36	6.53E-03	0.011	0.81	0.102	0.49	3.28E-03	1.80	0.450
C00122	Fumarate	Fumarate	3.26	7.80E-03	0.013	0.92	0.070	0.64	2.31E-03	1.21	0.714
C00078	Tryptophan	Tryptophan	3.21	8.68E-03	0.014	0.80	0.965	0.92	0.959	1.35	0.105
C00506	L-Cysteate	L-Cysteate	3.17	9.22E-03	0.015	0.90	0.109	3.95	4.56E-03	3.37	0.057
C02732	Peptide tryptophan	NA	3.13	0.010	0.016	0.83	0.067	0.76	0.038	1.44	0.784
C00214	Thymidine	Thymidine	2.88	0.016	0.025	0.98	0.238	1.94	0.185	3.75	0.170
C00491	Cystine	Cystine	2.84	0.017	0.027	0.91	0.741	1.00	0.772	0.04	0.122
C03573	2-Deoxy-alpha-D-glucoside	NA	2.80	0.018	0.028	0.89	0.204	0.97	0.341	1.06	0.658
C00354	D-Fructose 1-6-bisphosphate	Fructose 1,6-bisphosphate	2.71	0.022	0.033	1.74	0.803	0.21	0.023	0.92	0.810
C00062	Arginine	Arginine	2.63	0.025	0.038	0.94	0.612	0.97	0.655	2.20	0.500
C00318	L-Carnitine	L-Carnitine	2.58	0.028	0.041	0.91	0.030	1.04	0.934	1.56	0.999
C00021	S-Adenosyl-L-homocysteine	S-Adenosyl-L-homocysteine	2.55	0.029	0.043	1.13	0.996	1.44	0.187	1.63	0.233
C00606	3-Sulfino-L-alanine	3-Sulfino-L-alanine	2.52	0.031	0.045	1.02	0.275	1.90	0.013	2.81	0.110
C00316	Tripeptide	NA	2.49	0.032	0.047	1.35	7.62E-04	1.00	0.605	0.54	0.999
C08435	Triacanthine	NA	2.47	0.034	0.049	0.72	0.206	1.10	0.522	0.62	0.519
C00788	L-Adrenaline	L-Adrenaline	2.45	0.035	0.050	0.70	0.147	1.41	0.341	3.18	0.358
C00475	Cytidine	Cytidine	2.45	0.035	0.050	0.86	0.289	0.90	0.234	0.84	0.485

Supplementary Table S2. Metabolic pathways of greatest enrichment in the liver, 24 hours after AKI. Statistically significant analytes detected in the liver, 24 hours after AKI, were used for metabolic pathway analyses and metabolite enrichment analysis via MetaboAnalyst 3.0. Compound names were identified and matched against the KEGG and SMPDB. Pathways were deemed significant if FDR ≤0.05.

Pathway Analysis: KEGG	Total	Expected	Hits	Raw p	FDR
Cysteine and methionine metabolism	33	1.96	10	9.93E-06	4.65E-04
Aminoacyl-tRNA biosynthesis	48	2.85	12	1.11E-05	4.65E-04
Taurine and hypotaurine metabolism	8	0.47	5	3.22E-05	9.01E-04
Arginine biosynthesis	14	0.83	6	7.60E-05	1.60E-03
Glycine, serine and threonine metabolism	33	1.96	8	4.61E-04	7.75E-03
Arginine and proline metabolism	38	2.26	8	1.27E-03	1.535E-02
Pentose phosphate pathway	22	1.31	6	1.28E-03	1.535E-02
Glutathione metabolism	28	1.66	6	4.83E-03	5.074E-02
**Pathway Analysis: SMPDB**					
Warburg Effect	49	4.47	14	4.89E-05	4.84E-03
Glycolysis	20	1.82	8	1.79E-04	8.84E-03
Pentose Phosphate Pathway	27	2.46	9	3.49E-04	1.152E-02
Arginine and Proline Metabolism	48	4.38	12	7.14E-04	1.394E-02
Catecholamine Biosynthesis	14	1.28	6	8.07E-04	1.394E-02
Gluconeogenesis	30	2.74	9	8.45E-04	1.394E-02
Carnitine Synthesis	16	1.46	6	1.85E-03	2.615E-02
Urea Cycle	23	2.10	7	3.04E-03	3.758E-02
**Enrichment Analysis: KEGG**					
Aminoacyl-tRNA biosynthesis	48	2.89	11	7.50E-05	6.30E-03
**Enrichment Analysis: SMPDB**					
Warburg Effect	58	5.27	14	3.52E-04	1.520E-02
Glycine and Serine Metabolism	59	5.36	14	4.28E-04	1.520E-02
Arginine and Proline Metabolism	53	4.81	13	4.96E-04	1.520E-02
Pentose Phosphate Pathway	29	2.63	9	6.22E-04	1.520E-02
Glycolysis	25	2.27	8	1.02E-03	1.990E-02
Taurine and Hypotaurine Metabolism	12	1.09	5	2.63E-03	3.540E-02
Gluconeogenesis	35	3.18	9	2.76E-03	3.540E-02
Urea Cycle	29	2.63	8	2.97E-03	3.540E-02
Methionine Metabolism	43	3.91	10	3.59E-03	3.540E-02
Spermidine and Spermine Biosynthesis	18	1.63	6	3.62E-03	3.540E-02

Supplementary Table S3. Analytes of greatest fold change in the liver, 24 hours after AKI. Fold change for statistically significant analytes in the liver, 24 hours after AKI, were calculated (AKI/sham) with MetaboAnalyst 3.0.

Compound	Metaboanalyst Nomenclature	FC (AKI/Sham)	p-value	BH FDR
Phosphocreatine	Phosphocreatine	53.68	6.80E-07	1.45E-05
2--3--Cyclic CMP	2',3'-Cyclic CMP	13.37	2.05E-09	2.89E-07
L-Citrulline	L-Citrulline	11.71	7.17E-07	1.45E-05
Creatinine	Creatinine	6.80	3.06E-03	8.98E-03
N-Acetyl-L-citrulline	N-Acetyl-L-citrulline	6.37	1.48E-04	7.74E-04
Creatine	Creatine	6.17	2.95E-06	4.08E-05
L-Homocitrulline	L-Homocitrulline	6.09	5.74E-06	6.23E-05
Methylenediurea	NA	6.03	2.09E-06	3.68E-05
5-Guanidino-2-oxopentanoate	5-Oxoarginine	5.62	2.43E-05	1.68E-04
D-Ribose	D-Ribose	4.49	1.68E-05	1.28E-04
L-Arabinose	L-Arabinose	4.49	1.73E-05	1.28E-04
5-6-Dihydrothymine	Dihydrothymine	4.03	5.84E-08	1.65E-06
L-Cysteate	L-Cysteate	3.95	4.63E-03	0.013
Pantothenol	Pantothenol	3.89	4.26E-05	2.61E-04
6-Thioxanthine 5--monophosphate	NA	3.18	4.61E-04	2.03E-03
(S)(+)-Allantoin	(S)(+)-Allantoin	3.15	3.18E-06	4.08E-05
Allantoate	Allantoate	2.88	3.42E-04	1.61E-03
Ornithine	Ornithine	2.63	7.17E-04	2.96E-03
Taurine	Taurine	2.35	1.62E-05	1.28E-04
Putrescine	Putrescine	2.35	1.26E-03	4.56E-03
S-Glutathionyl-L-cysteine	S-Glutathionyl-L-cysteine	2.16	5.68E-04	2.43E-03
Ethanolamine phosphate	Ethanolamine phosphate	2.06	1.42E-04	7.72E-04
Spermidine	Spermidine	1.97	1.49E-05	1.28E-04
3-Phosphonooxypyruvate	Phosphohydroxypyruvic acid	1.93	9.24E-09	4.34E-07
3-Sulfino-L-alanine	3-Sulfino-L-alanine	1.90	0.017	0.037
N6-Methyl-L-lysine	N6-Methyl-L-lysine	1.71	5.02E-03	0.013
Succinyl sulfathiazole	NA	1.66	8.47E-03	0.021
2-Hydroxyglutarate/Citramalate	2-Hydroxyglutarate	1.65	7.34E-04	2.96E-03
Dehydroascorbate	Dehydroascorbate	1.34	1.56E-03	4.99E-03
AMP	AMP	1.21	0.018	0.037
Pyruvate	Pyruvate	0.76	3.30E-03	9.49E-03
Citrate	Citrate	0.76	0.024	0.047
Peptide tryptophan	NA	0.76	0.023	0.046
Proline	Proline	0.75	0.028	0.054
N5-Methyl-L-glutamine	N-methyl-L-glutamic Acid	0.68	0.017	0.036
Glutamate	Glutamate	0.68	0.016	0.036
Cysteine	Cysteine	0.66	3.62E-03	0.010
Fumarate	Fumarate	0.64	1.16E-03	4.30E-03
sn-glycero-3-Phosphoethanolamine	1-Acyl-sn-glycero-3-phosphoethanolamine	0.64	7.75E-04	3.04E-03
Serine	Serine	0.60	4.48E-04	2.03E-03
Alanine	Alanine	0.60	0.010	0.025
cis-p-Coumarate	cis-p-Coumarate	0.59	2.15E-03	6.60E-03
Tyrosine	Tyrosine	0.56	9.98E-03	0.024
Aspartate	Aspartate	0.56	1.81E-03	5.68E-03
2/3-Phospho-D-glycerate	2,3-Diphosphoglyceric acid	0.56	2.90E-04	1.41E-03
2-Methyleneglutarate	2-Methyleneglutarate	0.55	6.13E-03	0.016
Succinate	Succinate	0.55	0.016	0.036
Glutathione	Glutathione	0.53	1.40E-03	4.78E-03
Cys-Gly	Cys-Gly	0.53	1.30E-03	4.60E-03

5-Oxoproline	5-Oxoproline	0.51	4.88E-03	0.013
Glutamine	Glutamine	0.51	7.06E-03	0.018
S-Adenosyl-L-methionine	S-Adenosyl-L-methionine	0.51	1.09E-03	4.16E-03
Lactate	Lactate	0.49	2.53E-03	7.60E-03
D-Glucose	D-Glucose	0.49	0.013	0.030
alpha-D-Glucosamine 1-phosphate	alpha-D-Glucosamine 1-phosphate	0.47	1.64E-05	1.28E-04
3-Oxalomalate	3-Oxalomalate	0.46	3.84E-05	2.46E-04
Sedoheptulose 1-phosphate	Sedoheptulose 1-phosphate	0.45	0.021	0.043
2-3-Bisphosphoglycerate	2,3-Diphosphoglyceric acid	0.45	7.13E-03	0.018
5-10-Methenyltetrahydrofolate	5,10-Methenyltetrahydrofolic acid	0.44	0.021	0.043
D-Ribitol 5-phosphate	D-Ribitol 5-phosphate	0.44	1.26E-04	7.11E-04
3-Phospho-D-erythronate	4-Phospho-D-erythronate	0.40	0.011	0.027
Malate	Malate	0.35	4.30E-06	5.05E-05
D-Glucono-1-5-lactone 6-phosphate	6-Phosphonoglucono-D-lactone	0.30	1.46E-03	4.78E-03
sn-Glycerol 3-phosphate	sn-Glycerol 3-phosphate	0.28	2.09E-04	1.05E-03
3D-(3-5/4)-Trihydroxycyclohexane-1-2-dione	3D-(3,5/4)-Trihydroxycyclohexane-1,2-dione	0.28	2.51E-05	1.68E-04
5-Phospho-alpha-D-ribose 1-diphosphate	PRPP	0.28	2.52E-06	3.95E-05
D-Fructose	D-Fructose	0.24	0.028	0.054
D-Glyceraldehyde 3-phosphate	D-Glyceraldehyde 3-phosphate	0.22	1.25E-04	7.11E-04
D-Fructose 1-6-bisphosphate	Fructose 1,6-bisphosphate	0.21	0.014	0.031
Sodium glucuronate	Gluconic acid	0.20	5.83E-08	1.65E-06
6-Phospho-D-gluconate	6-Phospho-D-gluconate	0.19	1.42E-03	4.78E-03
D-Glucose 6-phosphate	D-Glucose 6-phosphate	0.12	9.69E-06	9.76E-05
N-Acyl-D-mannosaminolactone	NA	0.10	6.32E-09	4.34E-07

Supplementary Table S4. Analytes of greatest fold change in the kidney, 24 hours after AKI. Fold change for statistically significant analytes in the kidney, 24 hours after AKI, were calculated (AKI/sham) with MetaboAnalyst 3.0.

Compound	Metaboanalyst Nomenclature	FC (AKI/Sham)	p value	BH FDR
N-Acetyl-L-citrulline	N-a-Acetylcitrulline	24.92	1.22E-03	4.22E-03
N-Amidino-L-aspartate	Guanidinosuccinic acid	15.36	3.03E-08	1.34E-06
Creatinine	Creatinine	4.46	1.47E-02	3.33E-02
L-Citrulline	Citrulline	4.15	5.02E-09	4.44E-07
Homomethionine	Homomethionine	3.76	8.47E-05	4.54E-04
2-Deoxy-alpha-D-glucoside	deoxyglucoside	3.63	1.44E-02	3.30E-02
D-Ribose	D-Ribose	3.56	4.28E-06	4.46E-05
L-Arabinose	L-Arabinose	3.56	4.28E-06	4.46E-05
Phosphocreatine	Phosphocreatine	3.16	5.14E-05	3.05E-04
Putrescine	Putrescine	2.99	1.13E-05	9.30E-05
4-Acetamidobutanoate	4-Acetamidobutanoic acid	2.93	1.16E-05	9.30E-05
5-Guanidino-2-oxopentanoate	2-Oxoarginine	2.90	9.11E-04	3.23E-03
L-Homocitrulline	Homocitrulline	2.82	6.07E-06	5.65E-05
N6-Methyl-L-lysine	N(6)-Methyllysine	2.79	2.60E-04	1.18E-03
Pyridoxal	Pyridoxal	2.62	8.39E-05	4.54E-04
Maltose	Maltose	2.25	2.02E-03	6.39E-03
Triacanthine	6-Amino-7-(3-dimethylallyl)purine	1.99	5.57E-03	1.41E-02
Methylenediurea	NA	1.97	1.24E-04	6.46E-04
Ornithine	Ornithine	1.90	3.56E-03	9.73E-03
Creatine	Creatine	1.88	4.37E-05	2.76E-04
3-Oxalomalate	3-Oxalomalate	1.77	2.27E-04	1.06E-03
Histidine	L-Histidine	1.73	1.25E-03	4.25E-03
Acetylcholine	Acetylcholine	1.72	4.69E-03	1.21E-02
N-Acetylneuraminate	N-Acetylneuraminate	1.69	2.45E-05	1.67E-04
N5-Methyl-L-glutamine	N-methyl-L-glutamic Acid	1.64	8.86E-04	3.20E-03
D-Ribitol 5-phosphate	D-Ribitol 5-phosphate	1.63	3.57E-03	9.73E-03
Pyridoxamine	Pyridoxamine	1.54	1.75E-02	3.69E-02
N-Glycoloyl-neuraminate	N-Glycolylneuraminic acid	1.51	8.92E-03	2.19E-02
N-Carbamyl-L-glutamate	Carglumic acid	1.50	2.76E-03	8.00E-03
Arginine	L-Arginine	1.49	1.45E-03	4.77E-03
Dimethylglycine	Dimethylglycine	1.49	4.72E-03	1.21E-02
gamma-L-Glutamylputrescine	Gamma-glutamyl-L-putrescine	1.49	6.70E-03	1.67E-02
5-6-Dihydrothymine	Dihydrothymine	1.48	1.36E-02	3.17E-02
5-Hydroxyisourate	5-Hydroxyisourate	1.39	3.42E-03	9.61E-03
Dehydroascorbate	Dehydroascorbic acid	1.37	2.71E-02	5.40E-02
2-Oxoglutarate	Oxoglutaric acid	1.37	1.61E-02	3.51E-02
Oxalosuccinate	Oxalosuccinic acid	1.29	1.91E-04	9.13E-04
Pantothenate	Pantothenic acid	1.25	1.95E-02	3.98E-02
D-Glucose	D-Glucose	0.86	1.11E-02	2.66E-02
Lactate	L-Lactic acid	0.84	1.75E-02	3.69E-02
alpha-D-Ribose 1-phosphate	Ribose 1-phosphate	0.78	2.56E-03	7.54E-03
Taurine	Taurine	0.77	2.46E-03	7.38E-03
Guanine	Guanine	0.77	3.33E-03	9.49E-03
S-Adenosyl-L-homocysteine	S-Adenosylhomocysteine	0.76	1.52E-02	3.40E-02
g-Oxalo-crotonate	4-oxalocrotonate	0.75	2.37E-03	7.25E-03
5-Oxoproline	Pyroglutamic acid	0.74	1.56E-02	3.44E-02
Ethanolamine phosphate	O-Phosphoethanolamine	0.74	4.57E-04	1.88E-03
1-Hydroxy-2-aminoethylphosphonate	(2-Amino-1-hydroxyethyl)phosphonate	0.74	4.57E-04	1.88E-03
Methionine	L-Methionine	0.73	1.83E-02	3.77E-02
Sedoheptulose 1-phosphate	Sedoheptulose 1-phosphate	0.71	1.46E-03	4.77E-03
Phosphoserine	Phosphoserine	0.70	1.72E-02	3.69E-02

Choline	Choline	0.70	1.45E-04	7.34E-04
cis-p-Coumarate	4-Hydroxycinnamic acid	0.70	2.22E-03	6.88E-03
Guanosine	Guanosine	0.70	1.30E-02	3.07E-02
L-Carnitine	L-Carnitine	0.69	1.03E-05	9.09E-05
Tyrosine	L-Tyrosine	0.69	4.64E-03	1.21E-02
Glutamate	L-Glutamic acid	0.68	8.03E-04	2.96E-03
GMP	Guanosine monophosphate	0.65	6.69E-04	2.52E-03
gamma-L-Glutamyl-D-alanine	gamma-Glutamylalanine	0.64	6.00E-04	2.36E-03
Inosine	Inosine	0.63	1.78E-03	5.74E-03
Hydroxyacetone phosphate	Dihydroxyacetone Phosphate	0.62	5.19E-04	2.09E-03
Glycine	Glycine	0.62	2.08E-05	1.48E-04
gamma-Glutamyl-gamma-aminobutyrate	4-(Glutamylamino) butanoate	0.62	2.04E-02	4.11E-02
Shikimate 3-phosphate	Shikimate 3-phosphate	0.62	4.06E-04	1.80E-03
AMP	Adenosine monophosphate	0.61	4.29E-04	1.85E-03
Nicotinamide	Niacinamide	0.61	8.15E-05	4.54E-04
Peptide tryptophan	NA	0.61	3.71E-06	4.38E-05
trans-Homoaconitate	trans-Aconitic acid	0.61	4.71E-03	1.21E-02
NAD+	NAD	0.60	4.33E-05	2.76E-04
Pyruvate	Pyruvic acid	0.59	1.62E-04	7.96E-04
Glycerol 3-phosphate	Glycerol 3-phosphate	0.59	1.32E-05	1.01E-04
Hypoxanthine	Hypoxanthine	0.57	1.83E-05	1.35E-04
Cystine	L-Cystine	0.57	5.17E-05	3.05E-04
Xanthine	Xanthine	0.56	1.11E-02	2.66E-02
Adenine	Adenine	0.51	1.93E-06	2.63E-05
Adenosine	Adenosine	0.51	4.66E-06	4.58E-05
Poly-gamma-D-glutamate	Poly-g-D-glutamate	0.45	8.92E-08	2.63E-06
Valine	L-Valine	0.42	1.64E-06	2.42E-05
Cysteine	L-Cysteine	0.41	1.41E-06	2.27E-05
Guanidinoacetate	Guanidoacetic acid	0.35	4.12E-08	1.46E-06
Anthranilate	2-Aminobenzoic acid	0.34	3.35E-07	7.40E-06
4-Aminobenzoate	p-Aminobenzoic acid	0.34	3.35E-07	7.40E-06
L-Homocysteine	L-Homocysteine	0.34	1.33E-09	2.36E-07
5-L-Glutamyl-taurine	5-L-Glutamyl-taurine	0.33	4.84E-07	9.52E-06
gamma-L-Glutamyl-L-cysteine	gamma-Glutamylcysteine	0.31	2.86E-06	3.62E-05
Ectoine	Ectoine	0.31	6.02E-07	1.07E-05
Hypotaurine	Hypotaurine	0.19	8.12E-09	4.79E-07
Sorbitol	Sorbitol	0.04	6.59E-04	2.52E-03
L-gamma-Glutamyl-L-hypoglycin	Hypoglycin B	0.00	1.82E-02	3.77E-02

Supplementary Table S5. Metabolic pathways of greatest enrichment in the kidney, 24 hours after AKI. Statistically significant analytes detected in the kidney, 24 hours after AKI, were used for metabolic pathway analyses and metabolite enrichment analysis via MetaboAnalyst 3.0. Compound names were identified and matched against the KEGG and SMPDB. Pathways were deemed significant if FDR ≤0.05.

Pathway Analysis: KEGG	Total	Expected	Hits	Raw p	FDR
Glutathione metabolism	28	1.05	8	4.09E-06	2.25E-04
Arginine and proline metabolism	38	1.42	9	5.36E-06	2.25E-04
Glycine, serine and threonine metabolism	33	1.23	8	1.58E-05	4.41E-04
Purine metabolism	65	2.43	10	8.84E-05	1.55E-03
Arginine biosynthesis	14	0.52	5	9.54E-05	1.55E-03
Taurine and hypotaurine metabolism	8	0.30	4	1.11E-04	1.55E-03
Cysteine and methionine metabolism	33	1.23	7	1.42E-04	1.71E-03
Aminoacyl-tRNA biosynthesis	48	1.80	8	2.76E-04	2.89E-03
**Pathway Analysis: SMPDB**					
Glycine and Serine Metabolism	50	2.38	10	5.61E-05	5.55E-03
Purine Metabolism	63	3.00	10	4.36E-04	2.16E-02
Arginine and Proline Metabolism	48	2.28	8	1.28E-03	4.20E-02
Methionine Metabolism	39	1.86	7	1.70E-03	4.20E-02
**Enrichment Analysis: SMPB**					
Glycine and Serine Metabolism	59	3.11	15	6.72E-08	6.59E-06
Arginine and Proline Metabolism	53	2.79	12	6.94E-06	3.40E-04
Methionine Metabolism	43	2.27	10	3.60E-05	1.17E-03
Purine Metabolism	74	3.90	13	5.24E-05	1.26E-03
Urea Cycle	29	1.53	8	6.44E-05	1.26E-03
Cysteine Metabolism	26	1.37	7	2.31E-04	3.78E-03
Glucose-Alanine Cycle	13	0.69	5	3.16E-04	4.42E-03
Glutathione Metabolism	21	1.11	6	4.78E-04	5.21E-03
Betaine Metabolism	21	1.11	6	4.78E-04	5.21E-03
Glutamate Metabolism	49	2.58	9	6.42E-04	6.29E-03
Ammonia Recycling	32	1.69	7	9.24E-04	8.23E-03
Alanine Metabolism	17	0.90	5	1.29E-03	1.05E-02
Taurine and Hypotaurine Metabolism	12	0.63	4	2.50E-03	1.88E-02
Carnitine Synthesis	22	1.16	5	4.48E-03	3.14E-02
Gluconeogenesis	35	1.85	6	8.05E-03	5.26E-02
**Enrichment Analysis: KEGG**					
Arginine and proline metabolism	38	1.39	9	4.25E-06	3.57E-04
Glutathione metabolism	28	1.02	7	3.83E-05	1.61E-03
Purine metabolism	65	2.37	10	6.97E-05	1.67E-03
Arginine biosynthesis	14	0.51	5	8.39E-05	1.67E-03
Taurine and hypotaurine metabolism	8	0.29	4	9.97E-05	1.67E-03
Glycine, serine and threonine metabolism	33	1.20	7	1.20E-04	1.68E-03
Aminoacyl-tRNA biosynthesis	48	1.75	8	2.28E-04	2.73E-03
Cysteine and methionine metabolism	33	1.20	5	5.84E-03	6.13E-02

Supplementary Table S6. Fold change of liver analytes in 24-hour AKI vs sham from labeled carbon studies and respective p-values. Fold change and p-values were generated using Excel.

Compound	Mean Sham (n=8)	Mean AKI (n=8)	AKI vs Sham p-value	FC (AKI/Sham)
D-Glucose	49685413.75	30411301.25	9.0E-05	0.61
13C6 glucose	373135.25	1215966.225	0.02	3.26
D-Hexose 6-phosphate	32863308.75	25969764	0.25	0.79
13C6 hexose phosphate	111684.3913	368519.375	0.01	3.30
D-Fructose bisphosphate	786108.725	247103.2375	0.02	0.31
D-Glyceraldehyde 3-phosphate	1098420.075	691427.925	0.01	0.63
13C3 G3P	133515.425	68492.76	2.8E-04	0.51
Bisphosphoglycerate	94003.08	66982.275	0.11	0.71
Phosphoglycerate	2194400.375	2977098.625	0.15	1.36
13C3 phosphoglycerate	2709.906125	52914.29375	0.01	19.53
Phosphoenolpyruvate	227646.675	397017.2	0.05	1.74
13C3 PEP	1243.693	20179.645	5.5E-06	16.23
Pyruvate	3041905	2621194.25	0.12	0.86
Lactate	280607712.5	157377757.5	3.7E-05	0.56
13C3 lactate	4810750.125	5366278.5	0.53	1.12
Citrate	7844657	7187909.25	0.63	0.92
13C2 citrate	95725.57536	96004.46231	0.99	1.00
13C3 citrate	48753.93	44525.11875	0.75	0.91
Itaconate	1954841.625	2048185.988	0.70	1.05
Succinate	4978302.625	4807344.5	0.90	0.97
13C2 succinate	53374.07362	31686.62105	0.23	0.59
(S)-Malate	43292355	30085126.25	0.01	0.69
13C2 malate	428683.4882	333866.9081	0.07	0.78
13C3 malate	321705.075	175089.0556	0.01	0.54
Glycerol 1-phosphate	85794078.75	56087701.25	4.9E-04	0.65
13C3 glycerol phosphate	1932837	2095444.625	0.46	1.08
D-Glucono-1,5-lactone 6-phosphate	248979.3375	164908.8375	0.11	0.66
6-Phospho-D-gluconate	3613921.125	3158249.675	0.46	0.87
13C6 phosphogluconate	56506.48375	71002.68125	0.51	1.26
alpha-D-Ribose 1-phosphate	15148941.25	16686036.25	0.18	1.10
Sedoheptulose 1-phosphate	6772092.625	8778158.125	0.09	1.30
13C6 sedoheptulose phosphate	25331.41125	45554.62875	0.02	1.80
D-Erythrose 4-phosphate	443271.85	367978.6625	0.17	0.83
AMP	149494325	94419152.5	2.2E-03	0.63
13C2 AMP	164804.2925	110224.4344	0.17	0.67
13C5 AMP	105438.3113	25682.4075	4.9E-04	0.24
13C7 AMP	177880.6013	215158.47	0.66	1.21
GMP	16912266.25	16573397.5	0.80	0.98
13C2 GMP	67700.25513	47480.996	0.41	0.70
13C5 GMP	1822600.375	1024881.163	2.2E-03	0.56

13C7 GMP	218811.0125	163216.4388	0.12	0.75
IMP	19151026.25	12912746.25	4.4E-03	0.67
13C2 IMP	37367.37938	163300.1329	0.06	4.37
13C5 IMP	192768.875	249649.1663	0.18	1.30
13C7 IMP	138718.2963	168971.0563	0.54	1.22
ATP	1901182.375	1784423	0.66	0.94
13C2 ATP	28326.5122	20016.63648	0.37	0.71
ADP	16562393.75	15811830.38	0.66	0.95
13C2 ADP	137837.8106	158812.9025	0.37	1.15
13C5 ADP	5798567.875	3485400.088	0.02	0.60
13C7 ADP	167014.315	115216.8213	0.12	0.69
GDP	348233.675	300621.7375	0.25	0.86
13C2 GDP	643167.86	316254.152	0.03	0.49
13C5 GDP	148280.2625	80510.1425	0.01	0.54
Inosine	14992785	18435802.5	0.17	1.23
13C2 inosine	0	1808.805	0.33	na
13C5 inosine	4973829.5	2792533.125	1.1E-04	0.56
Adenosine	1737711	1939420.25	0.48	1.12
13C5 adenosine	131718.9075	66103.55	0.07	0.50
Guanosine	261852.845	332287.2875	0.22	1.27
13C2 guanosine	38273.58919	98468.74007	0.14	2.57
13C5 guanosine	54090.80125	26723.05875	0.02	0.49
Adenine	1376901.125	1412294.5	0.82	1.03
Xanthine	6957600	7210558	0.61	1.04
13C2 xanthine	38899.18125	89778.40705	0.01	2.31
Hypoxanthine	1931306.875	3005235.5	0.01	1.56
13C2 hypoxanthine	194935.5967	389332.6251	0.04	2.00
Urate	2857174.25	3332111.875	0.19	1.17
(S)(+)-Allantoin	3092128.125	6218538.625	0.03	2.01
CMP	1937559.375	1803170.325	0.56	0.93
UMP	31581596.25	29560623.75	0.29	0.94
L-Glutamate	45998328.75	32673736.25	0.01	0.71
13C2 Glu	479695.293	381170.6603	0.30	0.79
L-Glutamine	104202055	102142531.3	0.79	0.98
13C2 Gln	1503175.909	1415050.155	0.80	0.94
13C3 Gln	296499.5375	368777.0125	0.35	1.24
Glutathione (GSH)	1258322125	654999862.5	7.3E-05	0.52
13C2 GSH	526407.7125	155961.0488	0.13	0.30
13C3 GSH	1529210.25	1722428.325	0.70	1.13
13C4 GSH	43450.36125	91243.97625	0.36	2.10
L-Alanine	80701807.5	44249107.5	2.1E-04	0.55
Ala M+1	2864438.375	1511550.788	9.0E-04	0.53
13C2 Ala	243899.975	188636.1991	0.26	0.77
13C3 Ala	1304873.725	1202089.25	0.68	0.92

L-Serine	2243213	1628878.875	0.02	0.73
L-Aspartate	5352915.125	3902073.5	0.01	0.73
Asp M+1	301459.1	188974.415	0.01	0.63
Glycine	2108751.375	2135501.75	0.96	1.01
L-Cysteine	532475.2125	364776.625	0.02	0.69
13C3 Cys	244145.7625	206886.825	0.04	0.85
Glutathione disulfide	9001348.875	6541538.875	0.04	0.73
13C2 GSSG	31536792.21	11115011.14	5.2E-06	0.35
13C3 GSSG	7420124.875	2682260.875	1.0E-05	0.36
13C5 GSSG	460893.575	132733.0013	5.0E-05	0.29
13C4 GSSG	2059270.75	843434.0138	3.1E-04	0.41
(R)-S-Lactoylglutathione	332802.9	84089.95875	2.4E-04	0.25
S-Glutathionyl-L-cysteine	429771.4	558359.3	0.21	1.30
Cys-Gly	11682859.38	6088333.125	2.4E-04	0.52

Supplementary Table S7. Fold change of kidney analytes in 24-hour AKI vs sham from labeled carbon studies and respective p-values. Fold change and p-values were generated using Excel.

Compound	Mean Sham (n=8)	Mean AKI (n=8)	AKI vs Sham p-value	FC (AKI/Sham)
D-Glucose	39368133.75	24708590	3.5E-03	0.63
13C6 glucose	90420.8625	437666.2163	0.10	4.84
Hexose phosphate	1597996.125	1423640.875	0.11	0.89
13C6 Hexose phosphate	16689.60538	35577.34875	0.02	2.13
Fructose bisphosphate	43693.36625	42986.03063	0.95	0.98
13C6 Fructose bisphosphate	26485.22125	26934.78875	0.89	1.02
Glyceraldehyde 3-phosphate	321649.225	366835.25	0.34	1.14
13C3 Glyceraldehyde 3-phosphate	1062.586375	9381.343125	0.02	8.83
Bisphosphoglycerate	83594.405	81607.9	0.71	0.98
13C3 Bisphosphoglycerate	23164.12875	22998.24625	0.94	0.99
Phosphoglycerate	435671.375	375443.9875	0.43	0.86
13C2 phosphoglycerate	4047.7485	2550.71325	0.14	0.63
13C3 Phosphoglycerate	4006.914063	9583.404125	0.07	2.39
Phosphoenolpyruvate	32760.95625	37719.63125	0.54	1.15
13C2 Phosphoenolpyruvate	462606.5625	268652.5375	0.02	0.58
13C3 Phosphoenolpyruvate	13214.46713	8110.386125	0.17	0.61
Pyruvate	379019.6	420928.475	0.26	1.11
13C3 Pyruvate	11888.919	18713.29275	0.27	1.57
Lactate	30836592.5	24529091.25	0.11	0.80
13C2 Lactate	192645.465	189025.625	0.93	0.98
13C3 Lactate	998692.225	1541447.463	0.13	1.54
Citrate	2749550.75	2745423.25	0.99	1.00
13C2 Citrate	43612.81693	24226.31382	0.04	0.56
13C3 Citrate	9723.179375	7512.0985	0.44	0.77
13C4 Citrate	12165.07575	54018.4325	2.3E-04	4.44
13C5 Citrate	6081.408125	8379.4335	0.15	1.38
2-Oxoglutarate	125876.9375	186045.675	6.1E-04	1.48
Itaconate	747185.4875	1204940.013	1.3E-04	1.61
Succinate	8121901.375	5404494.5	1.4E-03	0.67
13C2 Succinate	99547.60769	50632.61651	0.01	0.51
13C3 Succinate	33067.2375	18846.59188	0.07	0.57
Fumarate	2379131.875	1633578	1.6E-03	0.69
13C2 Fumarate	10062.26019	4729.942026	0.03	0.47
Malate	16543746.25	10961618.13	5.2E-04	0.66
13C2 Malate	210126.8948	128823.7837	0.02	0.61
13C3 Malate	52577.155	41278.8845	0.40	0.79
Glycerol 1-phosphate	13797057.5	8617224.375	1.5E-05	0.62
13C3 Glycerol 1-phosphate	101771.695	180483.425	0.03	1.77
Glycerol 3-phosphate	14464562.5	15584487.5	0.46	1.08
13C2 Glycerol 3-phosphate	639703.2164	893180.8931	0.01	1.40

13C3 Glycerol 3-phosphate	50694.3975	58825.61625	0.24	1.16
6-Phospho-D-gluconate	14247.3225	14247.40875	1.00	1.00
13C6 Phosphogluconate	202521.15	191969.45	0.60	0.95
Ribose phosphate	2818051.25	2267250.25	0.09	0.80
Sedoheptulose 1-phosphate	462490.75	327234.6875	0.01	0.71
D-Erythrose 4-phosphate	49926.39625	54223.2175	0.55	1.09
AMP	22953152.5	15633931.25	6.5E-04	0.68
13C2 AMP	116466.0548	82360.58188	0.01	0.71
GMP	3210790.375	2114330.875	2.7E-04	0.66
13C2 GMP	25960.92521	21699.05891	0.16	0.84
M+5	14145.204	15807.3895	0.69	1.12
L-Glutamate	172900825	104583693.8	1.0E-04	0.60
13C2 Glutamate	2759282.504	1268406.859	6.5E-04	0.46
13C3 Glutamate	416597.0125	237177.4638	0.01	0.57
L-Glutamine	24315180	12091421.88	1.4E-04	0.50
13C2 Glutamine	358607.1805	120879.722	5.8E-05	0.34
13C3 Glutamine	54920.72375	10792.642	0.01	0.20
Glutathione (GSH)	62218233.75	86429196.25	0.24	1.39
13C2 GSH	602466.1716	366660.5504	0.19	0.61
13C3 GSH	312246.1375	232309.8125	0.27	0.74
13C4 GSH	18974.94225	13960.57	0.47	0.74
13C5 GSH	85169.42125	16989.55663	3.4E-06	0.20
L-Alanine	18723387.5	15919642.5	0.06	0.85
13C2 Ala	74319.98101	76905.9741	0.91	1.03
13C3 Ala	604099.4375	658854.2	0.64	1.09
L-Aspartate	13019103.75	9584560.125	0.01	0.74
13C2 Asp	195629.7901	113448.0206	0.01	0.58
13C3 Asp	46066.86625	32833.76375	0.22	0.71
L-Serine	2587417.75	2588554.25	0.99	1.00
Glycine	13183351.25	8562800	1.7E-04	0.65
L-Cysteine	54488810	32137577.5	2.0E-03	0.59
13C3 Cysteine	9933830.25	8227490.5	0.13	0.83
Cystine	1317604.913	533770.1	0.01	0.41
13C6 Cystine	29779.05125	21445.1	0.32	0.72
Glutathione disulfide	56517.41125	82622.3155	0.38	1.46
13C2 GSSG	377120.6583	711263.1194	0.15	1.89
13C3 GSSG	95361.40125	186099.7274	0.17	1.95
S-Glutathionyl-L-cysteine	927248.6875	487457.8375	0.01	0.53
13C2 Glutathionylcysteine	51202.53909	9440.448759	3.3E-03	0.18
Cys-Gly	1537613.25	3771543.125	4.8E-06	2.45
13C2 Cys-Gly	11026.602	105470.38	1.7E-07	9.57
